# CASC4/GOLM2 drives high grade serous carcinoma anoikis resistance through the recycling of EGFR

**DOI:** 10.1038/s41417-023-00703-1

**Published:** 2023-11-29

**Authors:** Jaidev Bapat, Tomomi M. Yamamoto, Elizabeth R. Woodruff, Lubna Qamar, Railey G. Mikeska, Katherine M. Aird, Zachary L. Watson, Lindsay W. Brubaker, Benjamin G. Bitler

**Affiliations:** 1 Cancer Biology Graduate Program, The University of Colorado Anschutz Medical Campus, Aurora, CO USA; 2 Department of Obstetrics & Gynecology, Division of Reproductive Sciences, The University of Colorado Anschutz Medical Campus, Aurora, CO USA; 3 Department of Pharmacology & Chemical Biology and UPMC Hillman Cancer Center, University of Pittsburgh School of Medicine, Pittsburgh, PA USA; 4 Department of Obstetrics & Gynecology, Division of Gynecologic Oncology, The University of Colorado Anschutz Medical Campus, Aurora, CO USA

**Keywords:** Ovarian cancer, Cell biology, Cancer microenvironment

## Abstract

Ovarian cancer is the deadliest gynecological malignancy, and accounts for over 150,000 deaths per year worldwide. The high grade serous ovarian carcinoma (HGSC) subtype accounts for almost 70% of ovarian cancers and is the deadliest. HGSC originates in the fimbria of the fallopian tube and disseminates through the peritoneal cavity. HGSC survival in peritoneal fluid requires cells to resist anoikis (anchorage-independent apoptosis). Most anoikis resistant mechanisms are dependent on microenvironment interactions with cell surface-associated proteins, such as integrins and receptor tyrosine kinases (RTKs). We previously identified the gene *CASC4* as a driver of anoikis resistance. CASC4 is predicted to be a Golgi-associated protein that may regulate protein trafficking to the plasma membrane, but CASC4 is largely uncharacterized in literature; thus, we sought to determine how CASC4 confers anoikis resistance to HGSC cells. Mining of publicly available ovarian cancer datasets (TCGA) showed that CASC4 is associated with worse overall survival and increased resistance to platinum-based chemotherapies. For experiments, we cultured three human HGSC cell lines (PEO1, CaOV3, OVCAR3), and a murine HGSC cell line, (ID8) with shRNA-mediated CASC4 knockdowns (CASC4 KD) in suspension, to recapitulate the peritoneal fluid environment in vitro. CASC4 KD significantly inhibited cell proliferation and colony formation ability, and increased apoptosis. A Reverse Phase Protein Assay (RPPA) showed that CASC4 KD resulted in a broad re-programming of membrane-associated proteins. Specifically, CASC4 KD led to decreased protein levels of the RTK Epidermal Growth Factor Receptor (EGFR), an initiator of several oncogenic signaling pathways, leading us to hypothesize that CASC4 drives HGSC survival through mediating recycling and trafficking of EGFR. Indeed, loss of CASC4 led to a decrease in both EGFR membrane localization, reduced turnover of EGFR, and increased EGFR ubiquitination. Moreover, a syngeneic ID8 murine model of ovarian cancer showed that knocking down CASC4 leads to decreased tumor burden and dissemination.

## Introduction

Epithelial ovarian cancer (EOC) is the deadliest gynecological malignancy [1], and the fifth leading cause of cancer related deaths in women in the United States [2]. The high grade serous carcinoma (HGSC) subtype accounts for about 70% of all EOC cases and is considered to be the deadliest; this is due to low early detection rates, which are attributed to the fact that patients typically present symptoms at later stages (III/IV) [2–6]. Standard of care for EOC is surgical debulking followed (and sometimes preceded) by platinum-based chemotherapies [7, 8]. However, the median progression free survival for EOC patients who present at advanced stages is about 18 months [1], thus highlighting a need to understand initial drivers of HGSC.

Most HGSC does not primarily originate from the ovary epithelium, but from the fallopian tube epithelium (FTE). The following is the most widely accepted model for HGSC development: Serous tubal intraepithelial carcinoma (STIC) lesions develop from FTE cells at the fimbriated end of the fallopian tube. STIC-associated cells detach from the epithelium and disseminate through the peritoneal fluid to colonize distant sites, including the omentum and ovaries [9, 10]. The dissemination process requires HGSC cells to survive anoikis, or apoptosis caused by loss of attachment between the extracellular matrix (ECM) and integrins [11]. In lung, colon, and breast cancers, anoikis resistance is mediated numerous factors, including an increase in receptor tyrosine kinase (RTK) pro-survival signaling [12–16]. Previous research from others and our lab have implicated several disparate mechanisms of anoikis resistance including NOTCH3, components of TGF-β signaling, chromobox protein homolog 2 (CBX2, an epigenetic reader), and Carnitine palmitoyltransferase 1A (CPT1A, involved in fatty acid oxidation) as promoting HGSC anoikis resistance [17–19], yet, there remains a knowledge gap in understanding specific mechanisms driving anoikis resistance in HGSC.

To address this, we published an unbiased genome-wide CRISPR/Cas9 screen followed by a transcriptomic analysis in in vitro models of disseminating HGSC to determine *novel* genes and pathways driving anoikis resistance [19]. One of the validated hits from the screen was the Cancer susceptibility candidate 4 (CASC4, also known as *GOLM2*) gene, which encodes a predicted Golgi-membrane protein [20, 21]. Apart from its predicted localization, little is known about CASC4’s functionality, in cancer or otherwise, apart from the fact that CASC4 mRNA undergoes alternative splicing [22], that shed and secreted CASC4 can induce a migratory phenotype in triple-negative breast cancer [21], and that CASC4 *suppresses* Tn-antigen mediated metastasis in breast cancer [23]. CASC4 has a paralog, GOLM1 (GOLPH2, GP73) [24], which is a driver of hepatocellular carcinoma through various pathways, including cholesterol-dependent recycling of members of the erythroblastic oncogene B (ERBB) family of RTKs, such as the epithelial growth factor receptor 1 (EGFR/ERBB1) and ERBB4 [25, 26]. In this study, we demonstrate that both CASC4 mRNA and protein are predictors of poor prognosis, and that loss of CASC4 expression promotes anoikis and upon culture in suspension reduces cancer cell viability. Mechanistically, loss of CASC4 differentially regulates secreted and cell surface-associated proteins. Specifically, we demonstrate that loss of CASC4 increases EGFR turnover and EGFR degradation, thus highlighting that inhibiting CASC4 may serve as a novel therapeutic target.

## Results

### High CASC4 is associated with poor clinical outcomes in ovarian cancer

**Fig. 1:**
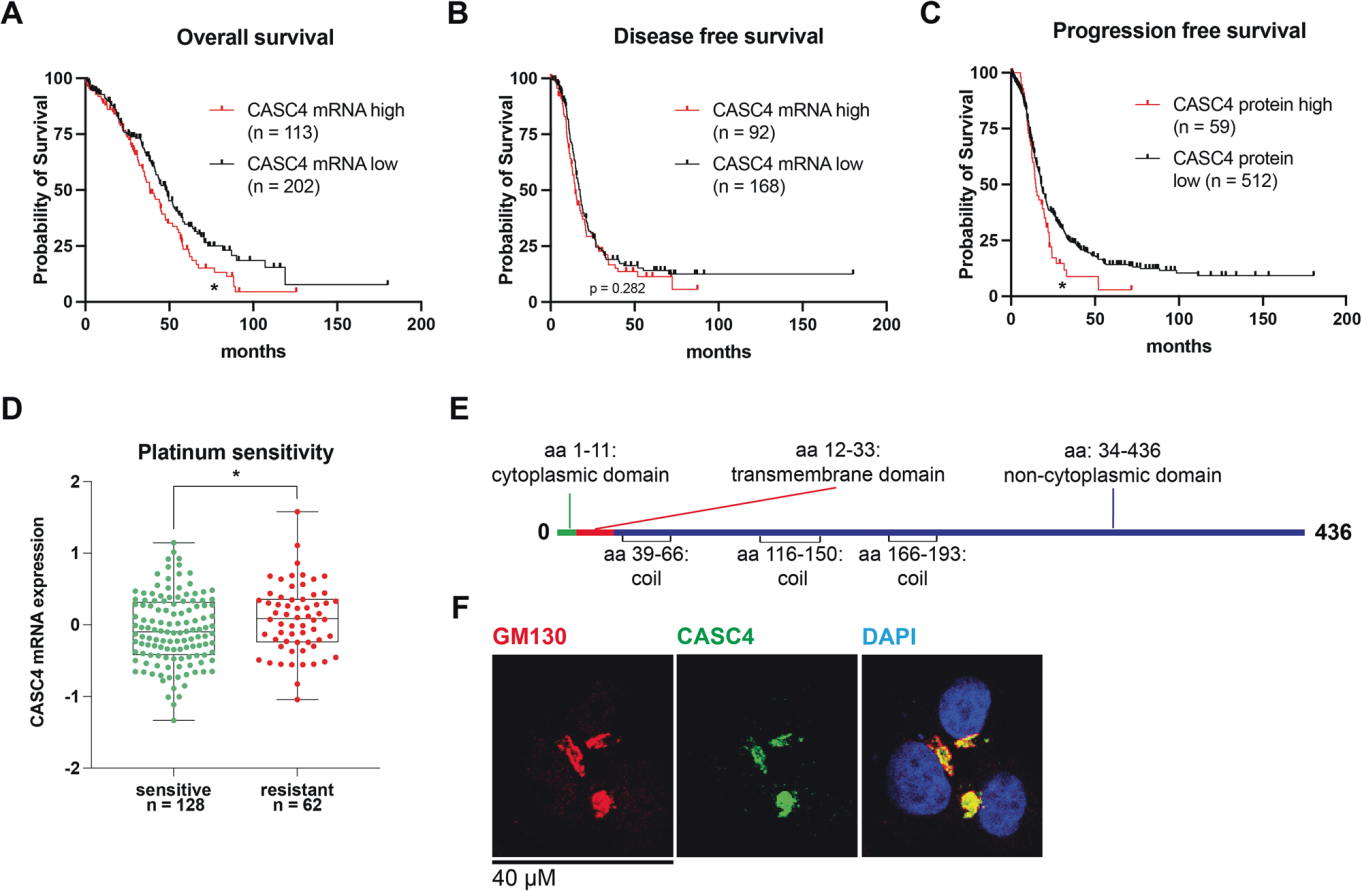
High CASC4 is associated with poor clinical outcomes in ovarian cancer. **A** Overall survival and (**B**) disease-free survival curves of patients with CASC4 RNA^high^ or ^low^ tumors. **C** Progression free survival curves of patients with CASC4 protein^high^ or ^low^ tumors. **D** Tumors resistant to platinum-based chemotherapies (such as cisplatin and carboplatin) express significantly higher CASC4 mRNA. **E** An InterProScan analysis shows that the CASC4 protein has a 22 amino acid-long transmembrane domain, which is indicative of human Golgi-localized transmembrane proteins [32]. **F** Immunofluorescence shows co-localization of CASC4 with Golgi marker GM130 in the PEO1 cell line. Statistical tests: **A**–**C** Log-rank (Mantel–Cox) test; **D** unpaired *t*-test. *p < 0.05, **p < 0.01, ***p < 0.001, ****p < 0.0001. Error bars show the standard error of measurement (SEM).

CASC4 was identified in a whole genome-wide CRISPR/Cas9 screen as being critical for HGSC cell survival in anchorage-independent suspension culture. Using canSAR Black [27] to examine the cancers associated with the highest CASC4 molecular score (which incorporates gene expression, mutations, and copy number alterations) showed that ovarian cancer had the second highest score (Fig. S1A). Next, through cBioPortal, we examined CASC4 mRNA and protein expression in a HGSC patient dataset published by The Cancer Genome Atlas (TCGA) [28–30]. Tumors with CASC4 mRNA levels above the mean were defined as CASC4 mRNA^high^ and below the mean were considered CASC4 mRNA^low^. Patients with CASC4 mRNA^high^ tumors exhibited worse overall survival outcomes (38.34 months vs. 48.29 months, Logrank *p* = 0.0265), but not disease-free survival outcomes (14.62 months vs. 17.51 months, Logrank *p* = 0.2822) (Fig. 1A, B). Similar stratification was performed on tumor mass spectrometry data, generating CASC4 protein^high^ and CASC4 protein^low^ populations; the CASC4 protein^high^ population exhibited significantly worse progression free survival outcomes (14.76 months vs. 18.05 months, Logrank *p* = 0.0216), compared to the low population (Fig. 1C). Also, patients with CASC4 mRNA^high^ tumors exhibited an increased resistance to standard-of-care platinum-based chemotherapies (e.g., cisplatin, carboplatin) compared to CASC4^low^ patients (Fig. 1D). Similar to anoikis resistance, an increase in chemotherapeutic resistance in CASC4^high^ patients suggests an increased utilization of pro-survival and/or anti-apoptotic pathways. CASC4 is predicted to be a Golgi transmembrane protein, based on its protein sequence and length of transmembrane domain [31, 32] (Fig. 1E). We confirmed the co-localization of CASC4 with GM130, a known Golgi marker, through immunofluorescence staining of ovarian cancer cells (Fig. 1E). Taken together, these data suggest that CASC4 is a Golgi-associated protein, and that its expression correlates to ovarian cancer progression, and worse clinical outcomes.

### Genetic inhibition of CASC4 hampers survival and proliferation in suspension conditions

**Fig. 2:**
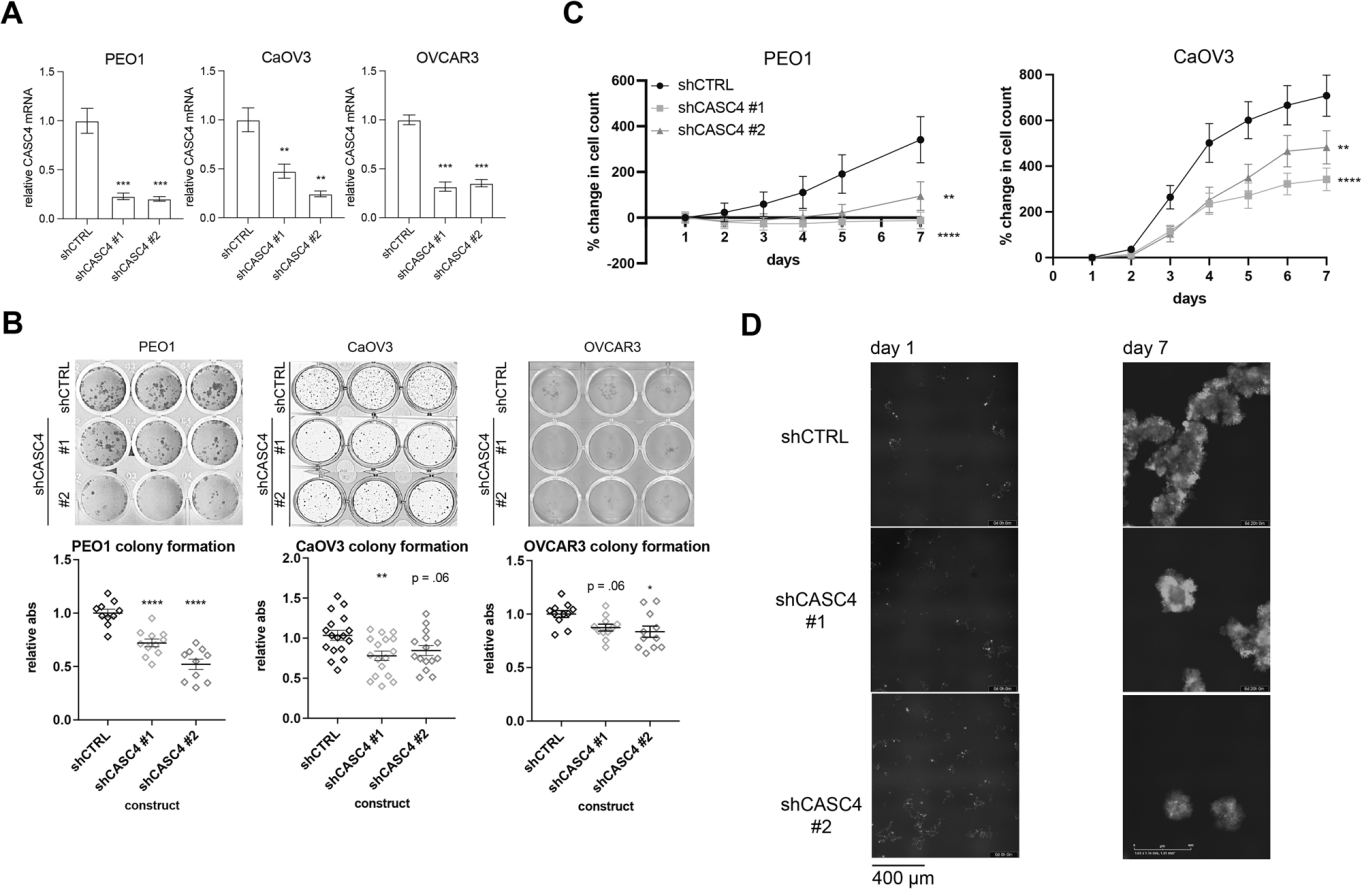
Genetic ablation of *CASC4* hampers survival and proliferation in suspension conditions. **A** Validation of shRNA-mediated CASC4 knockdowns (KD) in three different cell lines by qRT-PCR. **B** Colony formation assays performed with CASC4 KD cells. Cells were cultured in suspension for 2 days, then transferred to an adherent plate to allow for colony formation. Wells were stained with crystal violet and the absorbances were measured. Values represent the mean ± SEM of three independent experiments. **C**, **D** GFP-tagged cells were cultured in suspension for 7 days, and images were taken periodically using the Incucyte. GFP signal was used as a proxy for cell count. **C** Graph shows cell count over the course of the experiment. **D** Representative images of cells at day 1 and day 7. Values represent the mean ± SEM of three independent experiments. Statistical tests: (**A**, **B**) one-way ANOVA; (**C**) two-way ANOVA. *p < 0.05, **p < 0.01, ***p < 0.001, ****p < 0.0001. Error bars show the SEM.

Having drawn an association between CASC4 expression and HGSC malignancy and survival, we sought to determine if CASC4 was essential for driving anoikis resistance. We transduced CASC4 or control (CTRL) shRNAs into a panel of ovarian cancer cell lines including, PEO1, OVCAR3, and CaOV3. We confirmed knockdown (KD) through qRT-PCR (Fig. 2A). To characterize the effects of CASC4 KD on cell viability, we performed colony formation assays. shCTRL and shCASC4 cells were cultured in suspension for 2 days to allow for spheroid formation, as previously described [18], after which the cells were plated in adherent conditions, with colony formation serving as a surrogate for anoikis resistance. In suspension, CASC4 KD cells exhibited significantly fewer colonies, compared to the control cells (Fig. 2B). To characterize the effects of CASC4 KD on cell growth, GFP-tagged shCTRL and shCASC4 (#1 and #2) cells were cultured in suspension for 7 days, and cell spheroids were imaged via phase contrast and fluorescence. Over the 7-day period, CASC4 KD cells exhibited significantly fewer GFP-positive cells compared to the control cells (Fig. 2C, D). Anoikis is a form of programmed cell death; thus, to determine CASC4-dependent effects of suspension-induced cell death we performed flow cytometry for the negativity of a cell impermeable dye (DAPI) to identify live cells, that can exclude this dye. Compared to the control cells, CASC4 KD led to reduced live cells and increased dead cell counts in suspension conditions (Fig. S2A, B). In addition, compared to control KD, CASC4 KD cells culture in suspension and treated with cisplatin resulted in a significant decrease in live cells (Fig. S2C). These data demonstrate that the loss of CASC4 both significantly inhibits anchorage-independent survival, and improves chemotherapy response.

### CASC4 KD promotes proteomic reprogramming and identifies potential downstream effectors of CASC4-mediated anoikis resistance

**Fig. 3:**
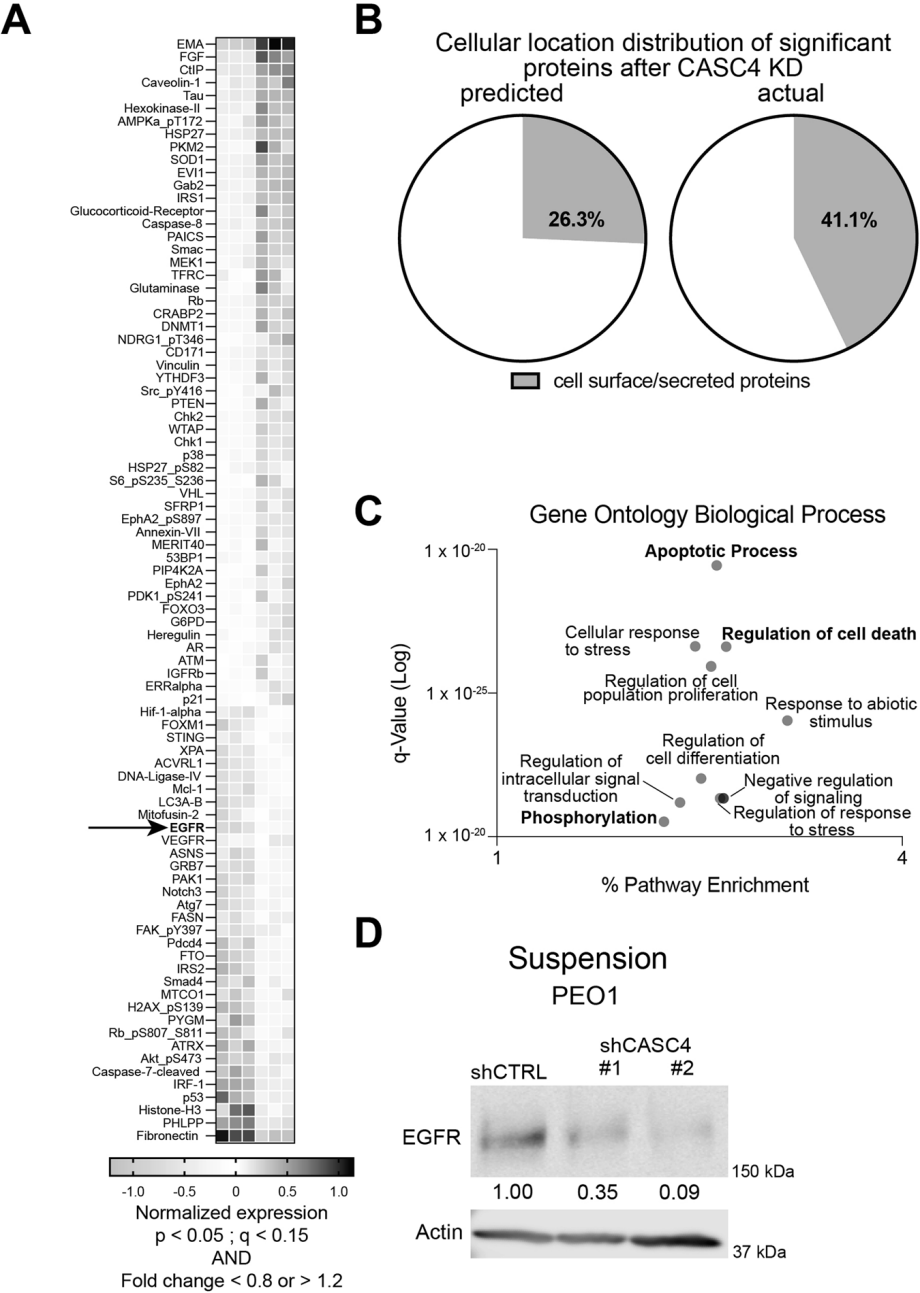
CASC4 KD promotes proteomic reprogramming and identifies potential downstream effectors of CASC4-mediated anoikis resistance. **A** A reverse phase protein array (RPPA) was performed on cells cultured in suspension for 2 days. Normalized, Log 2 median centered values of differentially expressed proteins (FDR < 0.15 with fold change <0.8 or fold change >1.2) are shown in the heatmap. **B** Graph comparing the frequencies of cell surface-localized or secreted proteins in the main RPPA panel, and in the set of signatures differently expressed after CASC4 KD. **C** Top 10 gene sets associated with proteins differentially altered after CASC4 KD. **D** Immunoblot confirming changes in EGFR after CASC4 KD. Densitometries show EGFR levels relative to actin, relative to shCTRL. Statistical tests: (**B**) Chi-squared test.

Having shown that the loss of CASC4 increases suspension-related cell death, we were subsequently interested in determining how CASC4 drives cell survival in suspension. Little is understood about CASC4 biology; however, a paralog of CASC4, GOLM1, is known to drive dissemination and apoptotic resistance in hepatocellular carcinoma cells through facilitating the recycling and membrane trafficking of RTKs, which then leads to increased activity of pro-survival and proliferative signaling pathways, such as the PI3K/AKT or MAPK pathways [26]. In order to identify cell signaling pathways potentially impacted by CASC4 KD in a relatively unbiased manner, we cultured PEO1 shCTRL and CASC4 KD cells in suspension for 2 days; followed by a reverse phase protein array (RPPA) analysis (Fig. 3A and Table S1). We noted 83 protein signatures differentially regulated. Notably, CASC4 KD lead to increased expression of apoptotic proteins SMAC, PTEN, RB, and FOXO3 (pro-apoptotic), and decreased expression of pS473 AKT, pY397 FAK, MCL-1 (anti-apoptotic). More importantly, while about 25.7% of the protein targets on the RPPA panel correspond to cell surface-localized proteins or secreted proteins, the loss of CASC4 significantly enriched for these proteins (25.7% vs. 42.9%, *p* = 0.004) (Fig. 3B), suggesting that CASC4 has a regulatory role in trafficking of cell surface-localized proteins or secreted proteins. Supporting this finding, a gene set enrichment analysis (GSEA) performed on ovarian cancer tumor RNA-seq data [28–30], shows that CASC4^high^ HGSC patients’ tumors are enriched for a post-Golgi vesicle mediated transport gene set (Fig. S3A). Additionally, a GSEA of the RPPA data showed that proteins differentially expressed after CASC4 KD were significantly overlapped with gene sets involved in regulation of cell death (Fig. 3C). These data show that CASC4 may serve to regulate trafficking of the transport of membrane-associated proteins.

To determine the mechanisms by which CASC4 drives anoikis resistance, we focused on the RTK EGFR, which was found to be downregulated after CASC4 KD (Fig. S3B). EGFR is a well-characterized oncogene, and therapies targeting EGFR, from small-molecular inhibitors (e.g., gefitinib, erlotinib) to monoclonal antibodies (e.g., cetuximab), are currently approved by the United States Food and Drug Administration (FDA) as treatments for various cancers, including colorectal cancers, non-squamous cell lung cancers, and head and neck cancers [33], but not for any ovarian cancers. EGFR has been associated with increased malignancy in ovarian cancer [34, 35]; however, clinical trials using EGFR inhibitors as ovarian cancer treatments have had varying results, primarily due to recurrence and the lack of suitable biomarkers for predicting response to EGFR inhibition [36], highlighting a need to better understand what drives EGFR-mediated ovarian cancer malignancy. We, then, sought to draw an association between CASC4 expression and EGFR expression. We confirmed that CASC4 KD leads to a decrease in total EGFR protein levels in suspension (Fig. 3D). EGFR signals to multiple downstream pro-survival signaling pathways, such as the PI3K/AKT pathways. The RPPA panel showed that phosphorylation of AKT at S473 was depleted in the CASC4 KD cells (Table S1), and we confirmed that CASC4 KD leads to decreased pAKT (S473) levels after a 2-day suspension culture (Fig. S3C). We also showed that CASC4 mRNA levels do not correlate with EGFR mRNA levels (Fig. S3D), suggesting that CASC4 regulates EGFR levels in a post-transcriptional manner. These data show that CASC4 regulates EGFR expression at the protein level and is likely involved in regulating the protein trafficking of EGFR and also that of other proteins trafficked through the secretory pathway.

### Loss of CASC4 attenuates recycling and cell surface trafficking of EGFR

**Fig. 4:**
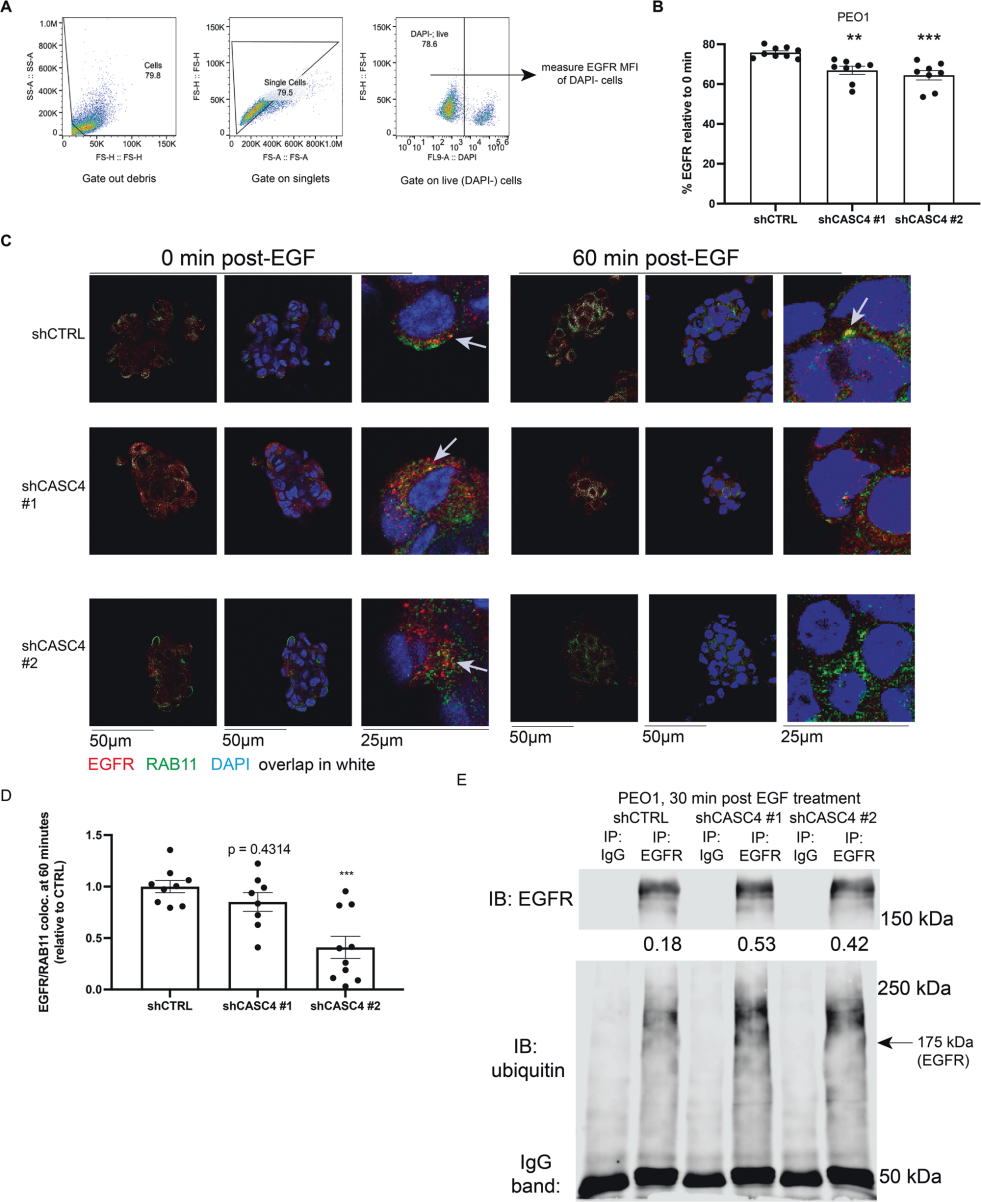
Loss of CASC4 hampers recycling and cell surface trafficking of EGFR. **A** Flow cytometry gating strategy. **B** Graph showing relative cell surface EGFR MFI from DAPI^−^ (live) PEO1 cells cultured in suspension for 3 h post-EGF treatment, normalized to PEO1 cells cultured in suspension for 0 h post-EGF treatment, relative to PEO1 shCTRL cells. Values represent the mean ± SEM of three independent experiments. **C** Representative images from IF experiments involving PEO1 shCTRL or shCASC4 spheroids treated with EGF and stained with DAPI (blue), EGFR (red), and RAB11 (green). Red/green co-localization areas represented in white. For each timepoint and construct: Left: overlap of RAB11/EGFR co-localization in white. Middle: RAB11/EGFR/DAPI. Right: Inset showing co-localization of RAB11/EGFR. White arrows show points of EGFR/RAB11 co-localization. **D** Quantification of (**C**). Bar graphs represent red/green co-localization areas per spheroid normalized to the number of nuclei, then normalized to the 0 min time point, then normalized to the shCTRL cells. **E** EGFR pull-down assay showing ubiquitination of EGFR protein from PEO1 shCTRL and CASC4 KD cells cultured in suspension and treated with EGF. Densitometries show ubiquitin levels relative to EGFR. Values represent the mean ± SEM of three independent experiments. Statistical tests: one-way ANOVA. *p < 0.05, **p < 0.01, ***p < 0.001, ****p < 0.0001.

Next, we sought to determine how CASC4 regulates EGFR protein levels, having already determined that the downregulation of EGFR was transcription-independent. We performed an EGFR recycling assay, to determine if CASC4 KD led to decreased retention of internalized EGFR. We treated cells with EGFR ligand EGF to activate and induce EGFR internalization, then letting the cells recover for either 0 or 3 h. Activation and phosphorylation of EGFR after EGF treatment was confirmed by immunoblot (Fig. S4A). Flow cytometry for cell surface EGFR was performed, and the EGFR levels at 3 h were normalized to those at 0 h, as an indication of protein retention, showing that CASC4 KD leads to decreased cell-surface localization EGFR post-EGF treatment, suggesting that CASC4 is involved in driving a recycling-like phenotype (Fig. 4A, B). EGFR, once internalized, is ubiquitinated and then degraded via lysosomes or proteosomes [25, 37], and the CASC4 paralog GOLM1 prevents lysosomal targeting of EGFR and facilitates recycling of EGFR back to the cell surface [26, 38]. Thus, we performed IF on EGF-treated PEO1 spheroids for EGFR and RAB11, a marker of recycling endosomes. Similar to our flow cytometry experiments, CTRL or CASC4 KD PEO1 cells cultured in suspension were treated with EGF. Spheroids were then fixed, and stained with DAPI, and antibodies against EGFR and RAB11. Confocal microscopy shows that CASC4 KD leads to decreased co-localization of EGFR with RAB11 at 60 min post-EGF treatment compared to 0 min (Fig. 4C, D). EGFR ubiquitination is required for both lysosome- and proteasome-mediated degradation of EGFR [39]. EGFR pull-down experiments showed that CASC4 KD led to increased ubiquitination of EGFR in the CASC4 KD cells (Fig. 4E). CASC4 overexpressing cells were also found to be more resistant to pharmacological EGFR inhibition by sapitinib (Fig. S5A, B). Our experiments show that CASC4 regulates the trafficking of internalized EGFR, thereby driving cancer cell survival.

### Casc4 KD leads to decreased tumor dissemination in vivo

**Fig. 5:**
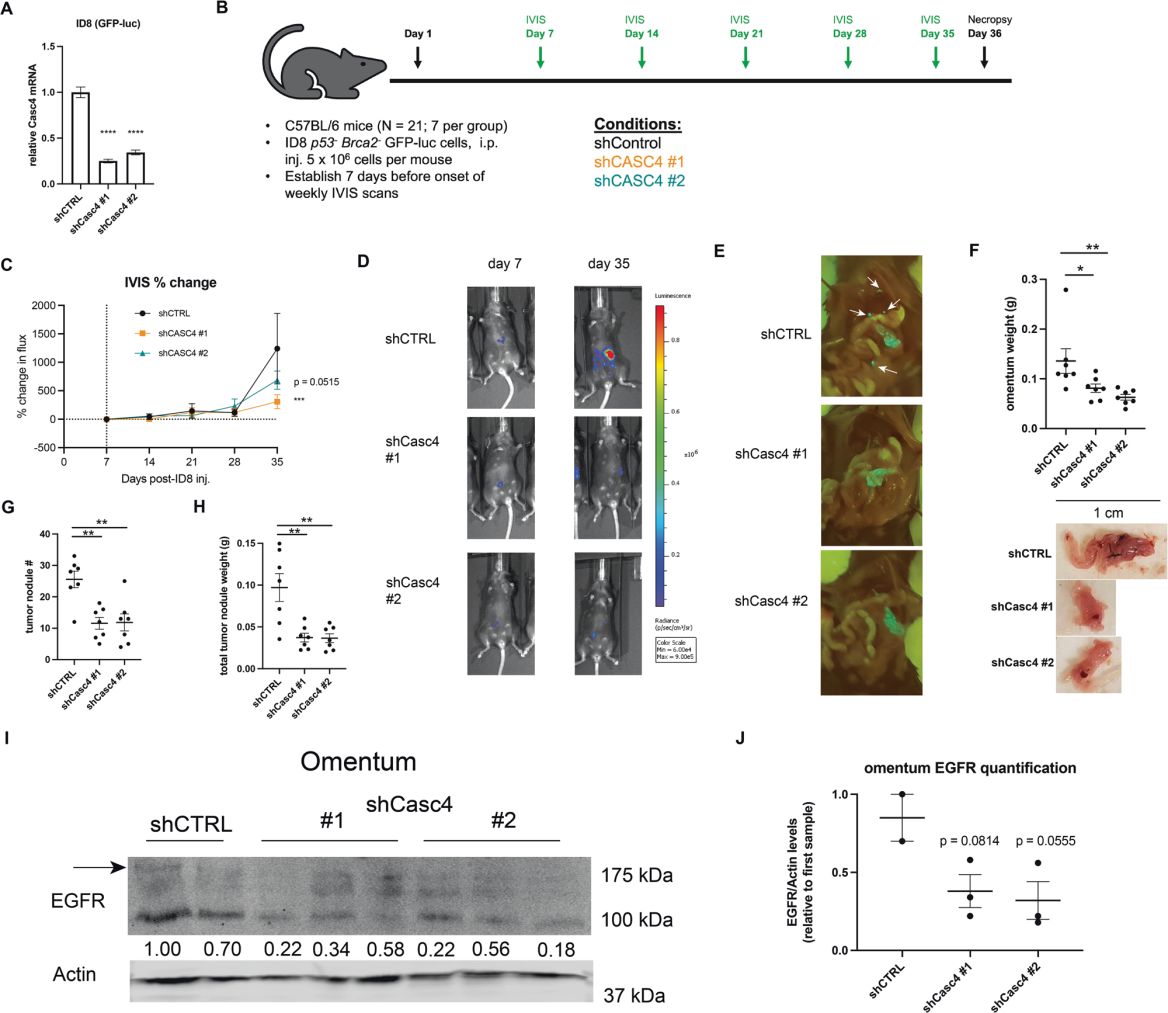
Casc4 KD leads to decreased tumor burden and ascites formation in vivo. **A** Validation of shRNA-mediated knockdown of Casc4 in ID8 cells (GFP, luciferase-tagged, Tp53-null, Brca2-null) by qRT-PCR. **B** Schematic of mouse experiment. **C** Graph showing percentage change in total flux over the experiment. **D** Representative luminescence images of mice 7 days and 35 days after injection. **E** Mouse peritoneal cavities exposed to UV light, showing locations of GFP^+^ tumors. **F** Top: Omentum weight of each mouse. Bottom: Representative omentum from the three groups. **G** Number of disseminated tumor nodules from each mouse. **H** Total weight of disseminated tumors from each mouse. **I** Immunoblot showing EGFR and actin protein levels in mouse omentum tissue. In the EGFR blot, the top band represents EGFR. Densitometries show EGFR levels relative to actin, relative to the first sample. **J** Densitometries for (**I**). Statistical tests: (**A**, **F**–**H**, **J**): one-way ANOVA, (**C**): two-way ANOVA. *p < 0.05, **p < 0.01, ***p < 0.001, ****p < 0.0001. Error bars show the SEM.

Having shown that CASC4 KD in human cell lines leads to decreased cell survival, proliferation, and EGFR recycling in suspension in vitro, we sought to establish whether modulating Casc4 drives similar phenotypes in vivo. To assess this, we used the syngeneic ID8 murine ovarian cancer cell line model (an EGFR-competent cell line [40]), which is considered an orthotopic model insofar as it recapitulates the tendency of human HGSC to target the omentum as a primary dissemination site. Human cell line models were not examined, as injecting human ovarian cancer cells into immunocompromised mice has demonstrated poor uptake and insufficient tumor formation [41]. We confirmed that Casc4 was knocked down in the ID8 shCasc4 cells, and that loss of Casc4 in ID8 cells leads to increased cell death in suspension in vitro (Fig. S6A), as is consistent with human HGSC lines. We intraperitoneally injected shCTRL or shCasc4 ID8 cells (*Trp53*-null, *Brca2*-null, GFP^+^ and luciferase-tagged) into female C57BL/6 mice (Fig. 5A, B). Measuring percent change in total flux (photons/s) through in vivo imaging showed that, on day 35 post-injection, mice with the Casc4 KD tumors exhibited decreased tumor burden, compared to those injected with the shCTRL cells (Fig. 5C, D). The total flux at day 35 was also significantly reduced in the mice injected with the Casc4 KD cells (Fig. S6B). Mice were sacrificed 5 weeks post-injection, and the omentum and disseminated tumors were harvested. Casc4 KD led to decreased gross GFP^+^ tumor formation throughout the peritoneal cavity (Fig. 5E). Mice injected with the Casc4 KD cells also had decreased omentum mass (Fig. 5F), decreased colonization of the omentum (Fig. 5G), and decreased tumor dissemination and decreased dissemination mass (Fig. 5H). Casc4 KD did not lead to increased apoptosis (cleaved caspase 3) or decreased proliferation (Ki67) of tumor cells at the omentum (Fig. S6C, D). However, omentum from mice from the CASC4 KD group had decreased EGFR protein levels (Fig. 5I, J). These experiments show that Casc4 regulates ovarian cancer cell survival, dissemination, and EGFR protein levels in vivo.

## Discussion

HGSC is the most common subtype of ovarian cancer and is also the deadliest. HGSC originates from the fallopian tube and disseminates through the peritoneal fluid before colonizing the ovaries; this dissemination process is one of the key factors in low early detection rates. To survive in peritoneal fluid, malignant cells must find a way to evade anoikis, or anchorage-independent cell death. While disparate drivers of HGSC anoikis resistance have been identified, no singular mechanism has been described.

**Fig. 6:**
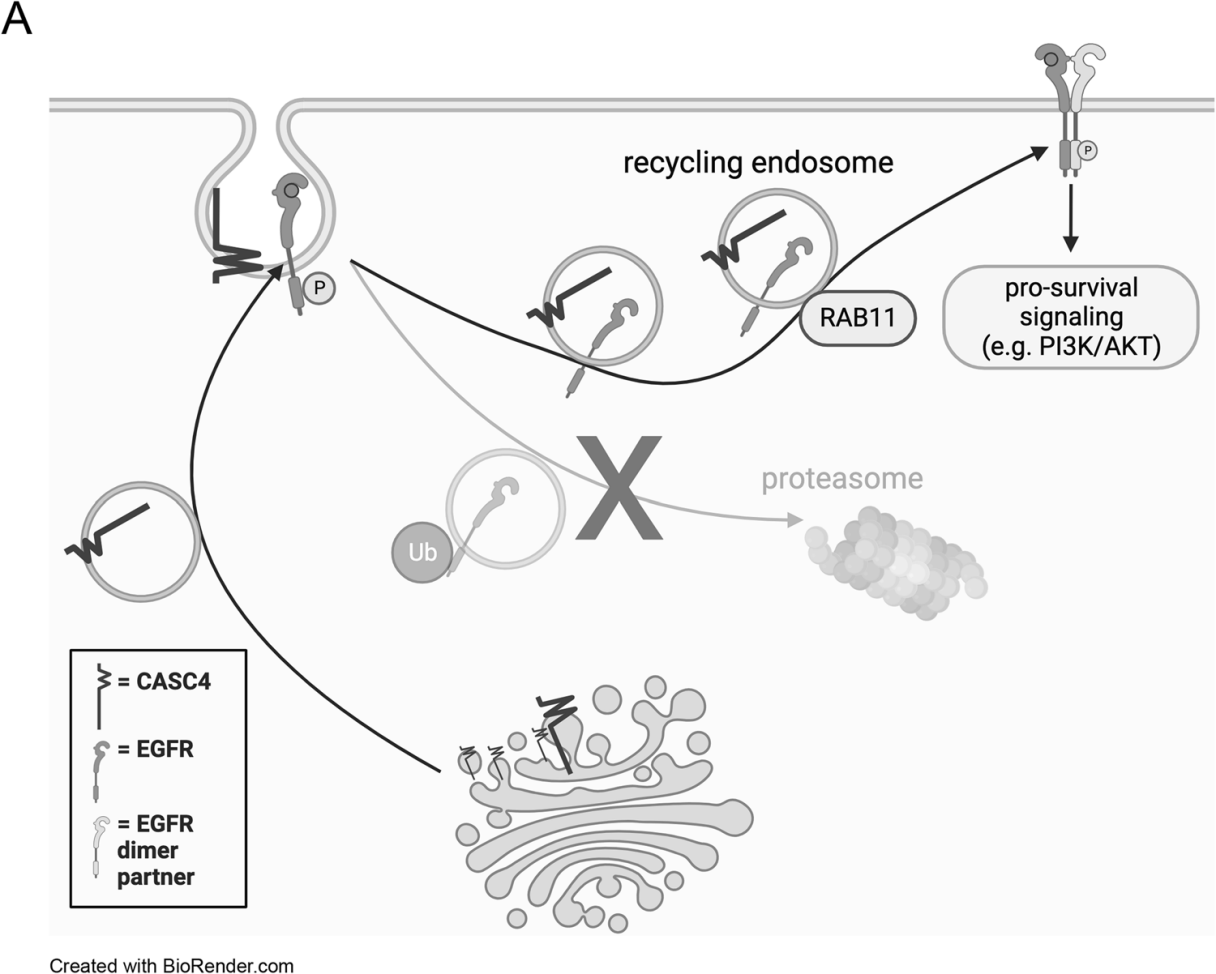
Working model of CASC4 function. CASC4 drives the recycling of internalized EGFR by promoting its localization to RAB11+ recycling endosomes and suppressing its ubiquitination. Recycling of EGFR to the cell surface allows for continued pro-survival signaling. Created using BioRender.

Our study shows that CASC4 regulates anoikis resistance in HGSC models, potentially through recycling of membrane-localized receptors, such as EGFR. We show that knocking down CASC4 significantly increases ovarian cancer cell death in suspension, decreases omental colonization, and inhibits disease dissemination in vivo. CASC4 was not found to regulate EGFR transcription, and was instead found to regulate the recycling of internalized EGFR, preventing its degradation by lysosomes, and promoting its trafficking back to the cell surface. While we focused on CASC4-mediated regulation of EGFR protein levels, there are several other differentially regulated proteins that likely contribute to the CASC4-dependent phenotypes, as identified through the RPPA experiment. Our research suggests that CASC4, similar to GOLM1, acts as a cargo adaptor, guiding internalized EGFR to a RAB11-involved trafficking mechanism (Fig. 6). We have also shown that CASC4 regulates EGFR ubiquitination.

A limitation to the study is that while we observed CASC4-mediated anoikis resistance in four independent models, most of the experiments focus on EGFR recycling in the PEO1 model. However, through analyzing TCGA data and gene signatures, there is a clear signal that CASC4 is involved in protein trafficking through the Golgi. Moreover, in the RPPA the significant dysregulation of membrane or secreted protein levels upon CASC4 KD, suggests that CASC4 can mediate anoikis resistance in HGSC through numerous downstream mechanisms. Our work delves into *how* CASC4 may regulate recycling of membrane proteins and, more importantly, establishes CASC4 as an indicator of malignancy in HGSC. Another limitation in our study regards our mouse model; however, the lack of differential cleaved caspase 3 expression in omentum sections, while important to note, does not invalidate our findings regarding CASC4 as a regulator of cell survival in suspension. The omentum IHC experiments only represent cells that have already successfully survived suspension, have disseminated to distant sites, and formed tumors.

Further elucidating mechanisms of CASC4-dependent anoikis resistance could help address numerous unique challenges posed by HGSC. The most common biomarker used to detect ovarian cancer is CA-125, a shed antigen found in patient sera [42]; however, it is now widely accepted that CA-125 levels alone do not divulge enough information regarding HGSC malignancy, and often correlate to advanced HGSC disease. Like CA-125, CASC4 is also shed extracellularly [21]; however, our research shows a mechanism through which CASC4 drives HGSC malignancy by facilitating its initial dissemination from the fallopian tube to the ovary, suggesting its potential suitability as a novel biomarker for detection of HGSC at earlier stages. Here, we have shown that CASC4 regulates trafficking of internalized EGFR. CASC4 may also be a biomarker for predicting response to EGFR inhibitors, which have otherwise seen mixed results as ovarian cancer treatments in clinical trials. Our experiments suggest that higher CASC4 levels are associated with increased resistance to EGFR inhibition. Additionally, while not confirmed in our study, CASC4 may be regulating the trafficking of other targetable membrane proteins, as suggested by the RPPA data. For example, CASC4 levels may be used to predict the efficacy of immune checkpoint inhibitors, which target cell surface proteins, whose presence at the plasma membrane may be CASC4-regulated; ovarian cancers, while highly immunogenic, have poor response rates to such therapies [43]. Ultimately, further research will need to be conducted, using additional models.

In summary, we have identified the Golgi-localized protein CASC4 as a driver of anoikis resistance in ovarian cancer through regulation of EGFR trafficking. Our study suggests that CASC4 may be a stronger and more viable biomarker for assessing HGSC malignancy, pending further research.

## Materials and methods

### Publicly available datasets

The Cancer Genome Atlas (TCGA) Ovarian Serous Cystadenocarcinoma dataset was accessed via cBioPortal (http://www.cbioportal.org) [28–30], for ovarian cancer patient RNA-seq data. This dataset was last accessed 31 January 2023. The TCGA PanCancer Atlas Ovarian Serous Cystadenocarcinoma dataset was accessed via cBioPortal, for ovarian cancer patient CPTAC data. This dataset was last accessed 6 Sept 2023. DepMap was used to correlate CASC4 dependencies or EGFR protein levels with sensitivities to EGFR inhibitors in ovarian cancer lines (https://depmap.org/portal) [44, 45]. DepMap was last accessed 30 May 2022. CASC4 molecular score was accessed through CanSar Black (https://cansarblack.icr.ac.uk) [46] on 30 May 2022.

### Cell culture

Human ovarian cancer cell lines PEO1 and OVCAR3 (obtained from the GTFB) and CaOV3 (obtained from Dr. Katherine Aird’s lab, University of Pittsburgh) were cultured in RPMI 1640 supplemented with 1% penicillin/streptomycin and 10% fetal bovine serum (referred to as “Complete RPMI”). Murine ovarian cancer cell line ID8 (obtained from Dr. Iain McNeish’s lab, Imperial College London) were cultured in high glucose DMEM supplemented with 1% penicillin/streptomycin, 4% fetal bovine serum, and 1% ITS (insulin-transferrin-selenium). ID8 cells were *Trp53-*null, *Brca2-*null, GFP^+^, and luciferase-tagged. Packaging cells (293FT; obtained from The University of Colorado Functional Genomics Shared Resource) were cultured in DMEM supplemented with 1% penicillin/streptomycin and 10% fetal bovine serum. For suspension experiments, cells were cultured as previously described [19]. Cells were authenticated at The University of Arizona via small tandem repeat analysis. Mycoplasma detection was performed regularly, using the MycoLookOut Mycoplasma PCR detection kit, from Sigma.

### CASC4 knockdowns and overexpression

All constructs were obtained from the University of Colorado Functional Genomics Facility (control shRNA: SHC001, pLKO.1-puro Empty Vector; RRID:Addgene_8453; shCASC4 #1: TRCN0000133832; shCASC4 #2: TRCN0000136384; empty vector: pLX304 control; CASC4 overexpression: ccsbBroad304_13019) and packaged using 293FT cells as previously described [18]. After 2 days, viruses were harvested from filtered 293FT media. Cells were transduced with the virus and treated with 1 mg/ml of hexadimethrine bromide (polybrene). After 24 h, media was refreshed. The cells used for experiments were a heterogenous mixture of antibiotic selected cells. For shRNA expressing cells, selection was performed with 1 μg/ml of puromycin. For CASC4 overexpression or empty vector expressing cells, selection was performed with 5 μg/ml of blasticidin.

### Quantitative reverse transcription polymerase chain reaction (qRT-PCR)

RNA extraction was performed using the RNAeasy Plus Mini Kit (Qiagen) as according to the manufacturer’s instructions. RT-qPCR was performed using the Luna Universal One-step qRT-PCR kit (New England BioLabs) on a BioRad thermocycler using primers for specific target transcripts. 18S rRNA was amplified as housekeeping genes. The following primer sequences were used:

18S (F: 5’- AACTTTCGATGGTAGTCGCCG-3’, R: 5’- CCTTGGATGTGGTAGCCGTTT-3’)

CASC4 (F: 5’-CAGAATCCTTCCAGTCCTCTTC-3’, R: 5’-CCTTGGTAGCCTGCTTTAGTAT-3’)

EGFR (F: 5’-AACACCCTGGTCTGGAAGTACG -3’, R: 5’- TCGTTGGACAGCCTTCAAGACC-3’).

Actb (F: 5’-TGTACCCAGGCATTGCTGAC-3’, R: 5’-AACGCAGCTCAGTAACAGTCC-3’)

Casc4 (F: 5’-TCCCCATGGGAAAGAACAACT-3’, R: 5’-GCTAACACAGGGGGCTTCTT-3’)

### EGFR recycling assay

Cells were plated in suspension in complete RPMI for 48 h, then switched over to serum-free RPMI for 24 h. Recombinant EGF (R&D systems; Cat. 236-EG-200) was then added to the cells to a final concentration of 20 ng/ml, and the cells were incubated on ice for 10 min. Cells were centrifuged for 5 min at 4 °C and 1000 rpm, resuspended in 1x PBS, centrifuged again for 5 min at 4 °C, then resuspended in serum-free RPMI, and incubated in suspension at 37 °C for the desired timepoints. Cells were then processed for flow cytometry analysis or immunofluorescence, as described below.

### Immunoblotting

For cell line experiments, cells were lysed with Radioimmunoprecipitation assay (RIPA) buffer (150 mM NaCl, 1% Triton X-100, 0.5% sodium deoxycholate, 0.1% SDS, 50 mM Tris pH 8.0, supplemented with complete EDTA-free protease inhibitors (Roche) and phosphatase inhibitors sodium fluoride (10 mM) and sodium orthovanadate (1 mM), briefly sonicated, and incubated on ice for 30 min. Lysates were then centrifuged at 4 °C, 15,000 rpm, for 10 min, and the supernatant was isolated. Protein concentrations in the supernatant were measured using the Pierce™ BCA Protein Assay Kit (Thermo Fisher), and the SpectraMaxM2e spectrophotometer (Molecular Devices). A similar procedure was followed for both human and mouse tissues, which were first homogenized with beads before sonication.

Protein lysates were run on SDS-Page gels and transferred onto methanol-activated PVDF membranes using the Trans-Blot Turbo Transfer System (Bio-Rad). Membranes were then dried at room temperature for 30 min, re-hydrated with methanol, and then incubated in blocking buffer (Intercept® Blocking Buffer (Li-Cor)) for 30 min and incubated with primary antibody overnight. Membranes were then washed thrice with TBS-T (50 mM Tris pH 7.5, 150 mM NaCl, 0.1% Tween-20), for 5 min each, incubated with secondary for 30 min, then washed thrice more with TBS-T. Membranes were imaged using the LI-COR Odyssey Imaging System. The antibodies used for immunoblotting can be found in Supplementary Table 2 (Table S2).

### Co-immunoprecipitation

EGFR recycling was performed as described above. Cells were cultured in suspension for 30 min after EGF was washed away, lysed using IP lysis buffer (50 mM Tris-HCl (pH 8.0), 1 mM EDTA, 0.5% Triton X-100, 150 mM NaCl, 10 mM *N-ethylmaleimide*, and the protease and phosphate inhibitors described above), and incubated on ice for 30 min. *N-ethylmaleimide* served as a deubiquitinase inhibitor. In total, 60 μl of Protein A magnetic beads (New England Biosciences) were washed with lysis buffer twice, and then used to pre-clear the protein lysate. In total, 1.35 mg of pre-cleared lysate was incubated with EGFR antibody (Cell Signaling Technologies, 7 μl of 4267S and 3 μl of 54359S) or IgG antibody (R&D Systems, AB-105-C) and rocked overnight at 4 °C. The next day, lysate-antibody mixtures were incubated with 30 μl of washed Protein A beads and rocked for 30 min at 4 °C. Supernatant was removed, and the beads were washed with lysis buffer and rocked for 5 min at 4 °C, thrice. Beads were then eluted with sample loading buffer, and boiled at 95 °C prior to the immunoblot. The antibodies used for IP can be found in Supplementary Table 2 (Table S2).

### Colony formation

In total, 25,000 cells were plated in suspension for 2 days, and then the media (containing the cells in suspension) was transferred to an adherent plate. After 7 days, cells were washed with PBS, fixed (10% acetic acid, 10% methanol in PBS) for 5 min, and then stained with crystal violet (0.4% crystal violet in ethanol). Plates were washed with deionized water and left to dry overnight. Crystal violet was then dissolved for 10 min in the fixation solution, and absorbance (at 590 nm) was measured using a SpectraMaxM2e spectrophotometer (Molecular Devices).

### Proliferation

In total, 15,000 cells were plated in suspension in 96 well plates, which were placed in a S3 Incucyte (Essen BioScience) for 7 days, with images of each well being taken every 4 h, at ×10 magnification. The GFP signal of each well was measured using the Incucyte Zoom (Essen BioScience) software, as a proxy for cell count.

### Flow cytometry

For flow cytometric detection of cell surface-localized EGFR, cells were grown in suspension for 2 days, then trypsinized for 5 min at 37 °C to obtain a single-cell suspension. Cells were then stained with a fluorophore-conjugated anti-EGFR antibody (see antibody chart for details) on ice in the dark for 30 min. Cells were washed with PBS and then stained with DAPI or Fixable Viability Stain 520 (BD Horizon™, Cat. # 564407) to differentiate between live and dead cells. Flow cytometry was performed using the Gallios 561 (Beckman Coulter) flow cytometer and data analysis was performed using FlowJo software. The EGFR intensity of DAPI^−^ (live cells) was measured. For flow cytometric detection of live cells, a similar protocol was performed, without the addition of the anti-EGFR antibody. The antibodies used for flow can be found in Supplementary Table 2 (Table S2).

### Immunofluorescence (IF)

Cells were grown in suspension for 2 days, washed with PBS, and fixed with 4% paraformaldehyde. Fixed cells were then spun onto charged slides using the Thermo Shandon Cytospin 2 (Thermo Fisher Scientific). Slides were washed again in PBS, permeabilized with 0.5% Triton X-100 for 5 min, washed with PBS three more times, and then blocked with blocking buffer (2% BSA in PBS) for 1 h. Slides were then incubated with primary antibody overnight at 4 °C. The next day, slides were washed with PBS thrice, incubated with DAPI and secondary antibodies (diluted in blocking buffer) for 30 min in the dark, and then washed thrice again in PBS, in the dark. Mounting medium (20 mg/mL O-phenylenediamine, dissolved in 1 M pH 8.5 Tris, and then diluted 1:10 in glycerol) was added to the slides, which were then covered with a glass coverslip and sealed with nail polish. The antibodies used for IF can be found in Supplementary Table 2 (Table S2). Confocal microscopy was performed using LSM780 (Zeiss).

Co-localization analysis was performed using the FIJI software. As the two proteins of interest were stained for using secondary antibodies conjugated to either AlexaFluor488 (represented as green) or Cy3 (represented as red), we performed thresholding to identify the “yellow” areas (as defined by a threshold value between 23–46), and normalized the “yellow” area to the number of cells (nuclei) for each image.

### Immunohistochemistry (IHC)

Immunohistochemistry on fixed mouse omentum for cleaved caspase 3 and Ki67 was performed, and slides were analyzed using the QuPath software, as previously described [47, 48]. The antibodies used for IHC can be found in Supplementary Table 2 (Table S2).

### Reverse phase protein array (RPPA)

One million PEO1 control or shCASC4 cells were cultured, each in triplicate, in suspension in a 6-well dish for 48 h, centrifuged, washed with PBS, and centrifuged again. Pellets were then flash-frozen at −80 °C and shipped to the MD Anderson Reverse Phase Protein Array Core Facility. Lysis and subsequent RPPA was performed by the core facility as outlined here (https://www.mdanderson.org/research/research-resources/core-facilities/functional-proteomics-rppa-core/education-and-references.html). Fold-changes (FC) for each antibody were calculated using the NormLinear values, relative to the average of the control (PEO1 shCTRL) cells. Significance was determined by performing a 2 tailed Student’s t-test between the NormLinear values for each antibody, with an FDR of 0.15.

### In vivo mouse experiments

All animal experiments were performed in accordance with the Guide for the Care and Use of Laboratory Animals and were approved by the University of Colorado IACUC (Protocol #00569). ID8 cells (*Trp53*-null, *Brca2*-null, GFP- and luciferase-tagged) were transduced with a control shRNA (CTRL), or one out of two shCasc4 constructs (labeled shCasc4 #1 and shCasc4 #2). In total, 5 × 10^6^ cells, suspended in 100 μl PBS, were injected intraperitoneally into 6–8 week-old female mice. Starting 7 days after initial cell injection, tumor burden was measured weekly using an In Vivo Imaging System, and analyzed using Live Imaging 4.0 software (PerkinElmer), as previously described [47]. Five weeks post-injection, mice were euthanized using a CO_2_ chamber followed by a cervical dislocation. The peritoneal cavity and harvested omentum tissue and tumors were exposed to UV light. Omentum and tumors were weighed. Each omentum was cut in half; one half was fixed in 4% paraformaldehyde for IHC, and the other was snap-frozen for immunoblot experiments.

### Statistical analysis and considerations

Statistical analyses and *p* value calculations were performed using GraphPad Prism 9 for MacOS. Quantitative data are expressed as mean ± standard error of the mean unless otherwise noted. Analysis of variance was used to identify significant differences in multiple comparisons. We performed *F*-tests for all tests. For all statistical analyses, the level of significance was set at 0.05. Outliers were identified using Grubbs’ outlier test with a power of 0.05, and excluded. For animal studies, no blinding or randomization was performed. A population size of 7 for each group was determined through a power analysis, using colony formation data as expected means.

### Supplementary information

Supplemental Figure and Table Legends

## Data Availability

All the data are presented in the manuscript and can be found within the figures and Supplementary files.

## References

[1] Jayson GC, Kohn EC, Kitchener HC, Ledermann JA (2014). Ovarian cancer. Lancet.

[2] Kuroki L, Guntupalli SR (2020). Treatment of epithelial ovarian cancer. BMJ.

[3] Lisio MA, Fu L, Goyeneche A, Gao ZH, Telleria C (2019). High-grade serous ovarian cancer: basic sciences, clinical and therapeutic standpoints. Int J Mol Sci.

[4] Koshiyama M, Matsumura N, Konishi I (2017). Subtypes of ovarian cancer and ovarian cancer screening. Diagnostics.

[5] Kim J, Park EY, Kim O, Schilder JM, Coffey DM, Cho CH (2018). Cell origins of high-grade serous ovarian cancer. Cancers.

[6] Suh-Burgmann EJ, Alavi M (2019). Detection of early stage ovarian cancer in a large community cohort. Cancer Med.

[7] Sato S, Itamochi H (2014). Neoadjuvant chemotherapy in advanced ovarian cancer: latest results and place in therapy. Ther Adv Med Oncol.

[8] Parmar MK, Ledermann JA, Colombo N, du Bois A, Delaloye JF, Kristensen GB (2003). Paclitaxel plus platinum-based chemotherapy versus conventional platinum-based chemotherapy in women with relapsed ovarian cancer: the ICON4/AGO-OVAR-2.2 trial. Lancet..

[9] Labidi-Galy SI, Papp E, Hallberg D, Niknafs N, Adleff V, Noe M (2017). High grade serous ovarian carcinomas originate in the fallopian tube. Nat Commun.

[10] Lee Y, Miron A, Drapkin R, Nucci MR, Medeiros F, Saleemuddin A (2007). A candidate precursor to serous carcinoma that originates in the distal fallopian tube. J Pathol.

[11] Frisch SM, Screaton RA (2001). Anoikis mechanisms. Curr Opin Cell Biol.

[12] Guadamillas MC, Cerezo A, Del Pozo MA (2011). Overcoming anoikis-pathways to anchorage-independent growth in cancer. J Cell Sci.

[13] Kim YN, Koo KH, Sung JY, Yun UJ, Kim H (2012). Anoikis resistance: an essential prerequisite for tumor metastasis. Int J Cell Biol.

[14] Paoli P, Giannoni E, Chiarugi P (2013). Anoikis molecular pathways and its role in cancer progression. Biochim Biophys Acta.

[15] Guha D, Saha T, Bose S, Chakraborty S, Dhar S, Khan P (2019). Integrin-EGFR interaction regulates anoikis resistance in colon cancer cells. Apoptosis.

[16] Huang RY, Wong MK, Tan TZ, Kuay KT, Ng AH, Chung VY (2013). An EMT spectrum defines an anoikis-resistant and spheroidogenic intermediate mesenchymal state that is sensitive to e-cadherin restoration by a src-kinase inhibitor, saracatinib (AZD0530). Cell Death Dis.

[17] Sawyer BT, Qamar L, Yamamoto TM, McMellen A, Watson ZL, Richer JK (2020). Targeting fatty acid oxidation to promote anoikis and inhibit ovarian cancer progression. Mol Cancer Res.

[18] Wheeler LJ, Watson ZL, Qamar L, Yamamoto TM, Post MD, Berning AA (2018). CBX2 identified as driver of anoikis escape and dissemination in high grade serous ovarian cancer. Oncogenesis.

[19] Wheeler LJ, Watson ZL, Qamar L, Yamamoto TM, Sawyer BT, Sullivan KD (2019). Multi-omic approaches identify metabolic and autophagy regulators important in ovarian cancer dissemination. iScience.

[20] Thul PJ, Akesson L, Wiking M, Mahdessian D, Geladaki A, Ait Blal H (2017). A subcellular map of the human proteome. Science.

[21] Duval S, Abu-Thuraia A, Elkholi IE, Chen R, Seebun D, Mayne J (2020). Shedding of cancer susceptibility candidate 4 by the convertases PC7/furin unravels a novel secretory protein implicated in cancer progression. Cell Death Dis.

[22] Anczukow O, Akerman M, Clery A, Wu J, Shen C, Shirole NH (2015). SRSF1-regulated alternative splicing in breast cancer. Mol Cell.

[23] Li R, Dong X, Chen S, Tan J, Chen X, Liu J (2023). Tn antigen promotes breast cancer metastasis via impairment of CASC4. Cell Biol Int.

[24] Stelzer G, Rosen N, Plaschkes I, Zimmerman S, Twik M, Fishilevich S (2016). The GeneCards suite: from gene data mining to disease genome sequence analyses. Curr Protoc Bioinformatics.

[25] Shao WQ, Zhu WW, Luo MJ, Fan MH, Li Q, Wang SH (2022). Cholesterol suppresses GOLM1-dependent selective autophagy of RTKs in hepatocellular carcinoma. Cell Rep.

[26] Ye QH, Zhu WW, Zhang JB, Qin Y, Lu M, Lin GL (2016). GOLM1 modulates EGFR/RTK cell-surface recycling to drive hepatocellular carcinoma metastasis. Cancer Cell.

[27] Mitsopoulos C, Di Micco P, Fernandez EV, Dolciami D, Holt E, Mica IL (2021). canSAR: update to the cancer translational research and drug discovery knowledgebase. Nucleic Acids Res.

[28] Cerami E, Gao J, Dogrusoz U, Gross BE, Sumer SO, Aksoy BA (2012). The cBio cancer genomics portal: an open platform for exploring multidimensional cancer genomics data. Cancer Discov.

[29] Gao J, Aksoy BA, Dogrusoz U, Dresdner G, Gross B, Sumer SO (2013). Integrative analysis of complex cancer genomics and clinical profiles using the cBioPortal. Sci Signal.

[30] Cancer Genome Atlas Research Network. (2011). Integrated genomic analyses of ovarian carcinoma. Nature.

[31] Jones P, Binns D, Chang HY, Fraser M, Li W, McAnulla C (2014). InterProScan 5: genome-scale protein function classification. Bioinformatics.

[32] Singh S, Mittal A (2016). Transmembrane domain lengths serve as signatures of organismal complexity and viral transport mechanisms. Sci Rep.

[33] Xu MJ, Johnson DE, Grandis JR (2017). EGFR-targeted therapies in the post-genomic era. Cancer Metastasis Rev.

[34] Wilken JA, Badri T, Cross S, Raji R, Santin AD, Schwartz P (2012). EGFR/HER-targeted therapeutics in ovarian cancer. Future Med Chem.

[35] Sheng Q, Liu J (2011). The therapeutic potential of targeting the EGFR family in epithelial ovarian cancer. Br J Cancer.

[36] Wang Q, Peng H, Qi X, Wu M, Zhao X (2020). Targeted therapies in gynecological cancers: a comprehensive review of clinical evidence. Signal Transduct Target Ther.

[37] Alwan HA, van Zoelen EJ, van Leeuwen JE (2003). Ligand-induced lysosomal epidermal growth factor receptor (EGFR) degradation is preceded by proteasome-dependent EGFR de-ubiquitination. J Biol Chem.

[38] Ferro E, Bosia C, Campa CC (2021). RAB11-mediated trafficking and human cancers: an updated review. Biology.

[39] Longva KE, Blystad FD, Stang E, Larsen AM, Johannessen LE, Madshus IH (2002). Ubiquitination and proteasomal activity is required for transport of the EGF receptor to inner membranes of multivesicular bodies. J Cell Biol.

[40] Lizotte PH, Hong RL, Luster TA, Cavanaugh ME, Taus LJ, Wang S (2018). A high-throughput immune-oncology screen identifies EGFR inhibitors as potent enhancers of antigen-specific cytotoxic T-lymphocyte tumor cell killing. Cancer Immunol Res.

[41] Karakashev S, Zhang RG (2021). Mouse models of epithelial ovarian cancer for preclinical studies. Zool Res.

[42] Charkhchi P, Cybulski C, Gronwald J, Wong FO, Narod SA, Akbari MR (2020). CA125 and ovarian cancer: a comprehensive review. Cancers.

[43] Borella F, Ghisoni E, Giannone G, Cosma S, Benedetto C, Valabrega G (2020). Immune checkpoint inhibitors in epithelial ovarian cancer: an overview on efficacy and future perspectives. Diagnostics.

[44] Corsello SM, Nagari RT, Spangler RD, Rossen J, Kocak M, Bryan JG (2020). Discovering the anti-cancer potential of non-oncology drugs by systematic viability profiling. Nat Cancer.

[45] Tsherniak A, Vazquez F, Montgomery PG, Weir BA, Kryukov G, Cowley GS (2017). Defining a cancer dependency map. Cell.

[46] Halling-Brown MD, Bulusu KC, Patel M, Tym JE, Al-Lazikani B (2012). canSAR: an integrated cancer public translational research and drug discovery resource. Nucleic Acids Res.

[47] McMellen A, Yamamoto TM, Qamar L, Sanders BE, Nguyen LL, Ortiz Chavez D (2022). ATF6-mediated signaling contributes to PARP Inhibitor Resistance in Ovarian Cancer. Mol Cancer Res.

[48] Bankhead P, Loughrey MB, Fernandez JA, Dombrowski Y, McArt DG, Dunne PD (2017). QuPath: open source software for digital pathology image analysis. Sci Rep.

