# Supplementary material for: CASC4/GOLM2 drives high grade serous carcinoma anoikis resistance through the recycling of EGFR
Source: Cancer Gene Ther. 2023 Nov 29;31(2):300–10. doi: 10.1038/s41417-023-00703-1 (PMC10874890; doi:10.1038/s41417-023-00703-1)
Supplement: Supplementary file 1 — Supplemental Figure and Table Legends [file 41417_2023_703_MOESM1_ESM.pdf]

SUPPLEMENTAL FIGURE AND TABLE LEGENDS

Figure S1, Bapat J, et al, 2022

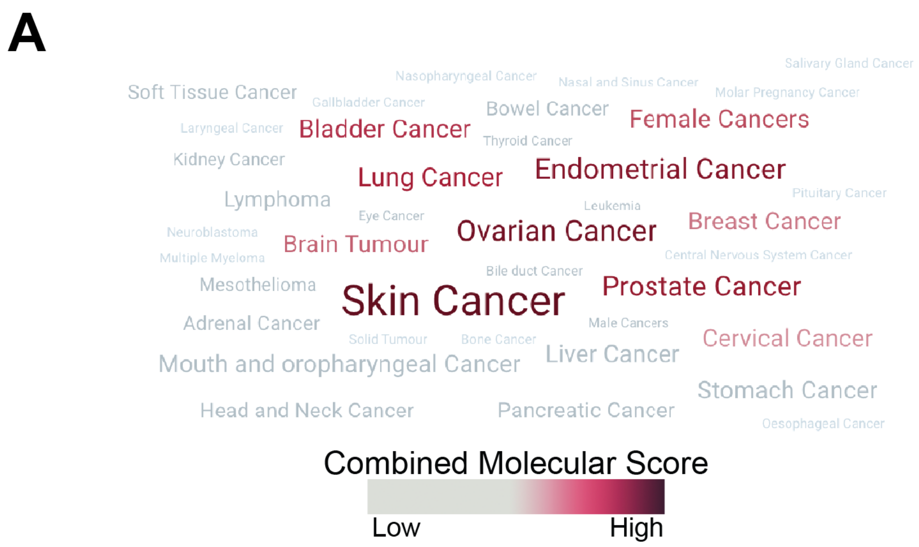

FIGURE S1. CASC4 is a Golgi-localized transmembrane protein. CanSar Black Combined Molecular Score for CASC4/GOLM2 across 38 cancer types.

Figure S2, Bapat J, et al, 2023

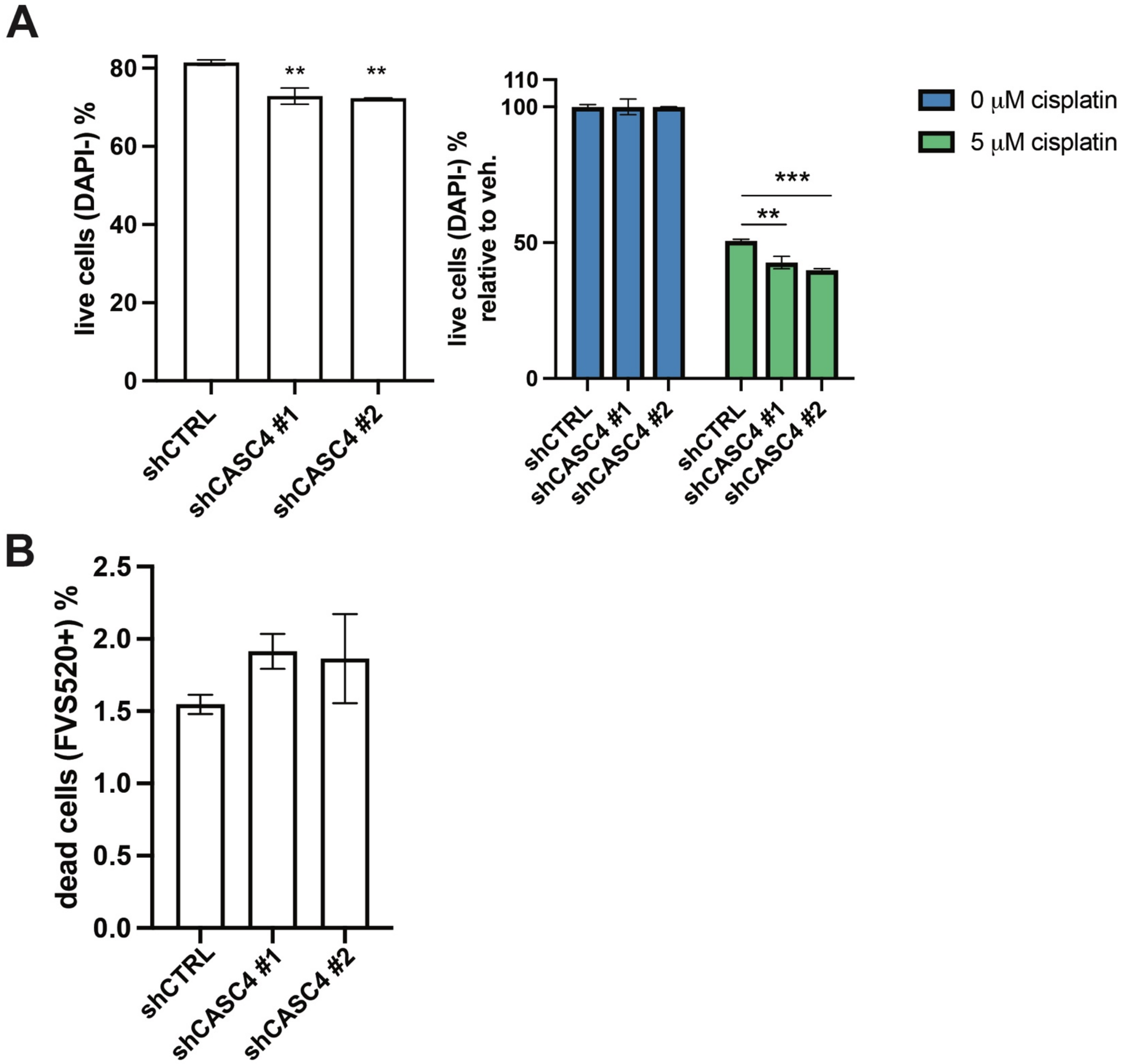

FIGURE S2. CASC4 KD leads to increased cell death in suspension and increased sensitivity to cisplatin. (A) Flow cytometry was performed on PEO1 cells expressing shCTRL or shCASC4 cultured in suspension and treated with the indicated doses of cisplatin for 2 days, gating on the DAPI^{-} (live) cells. Left: Live cell percentages in untreated cells. Right: Live cell percentages in cisplatin-treated cells, normalized to untreated cells. (B) CASC4 KD leads to increased staining of live/dead cell dye FVS520 (BD Biosciences). Statistical test: (A, B) one-way

ANOVA. $^{*}p < 0.05$, $^{**}p < 0.01$, $^{***}p < 0.001$, $^{****}p < 0.0001$. Values represent the mean ± SEM of 2 independent experiments.

Figure S3, Bapat J, et al, 2022

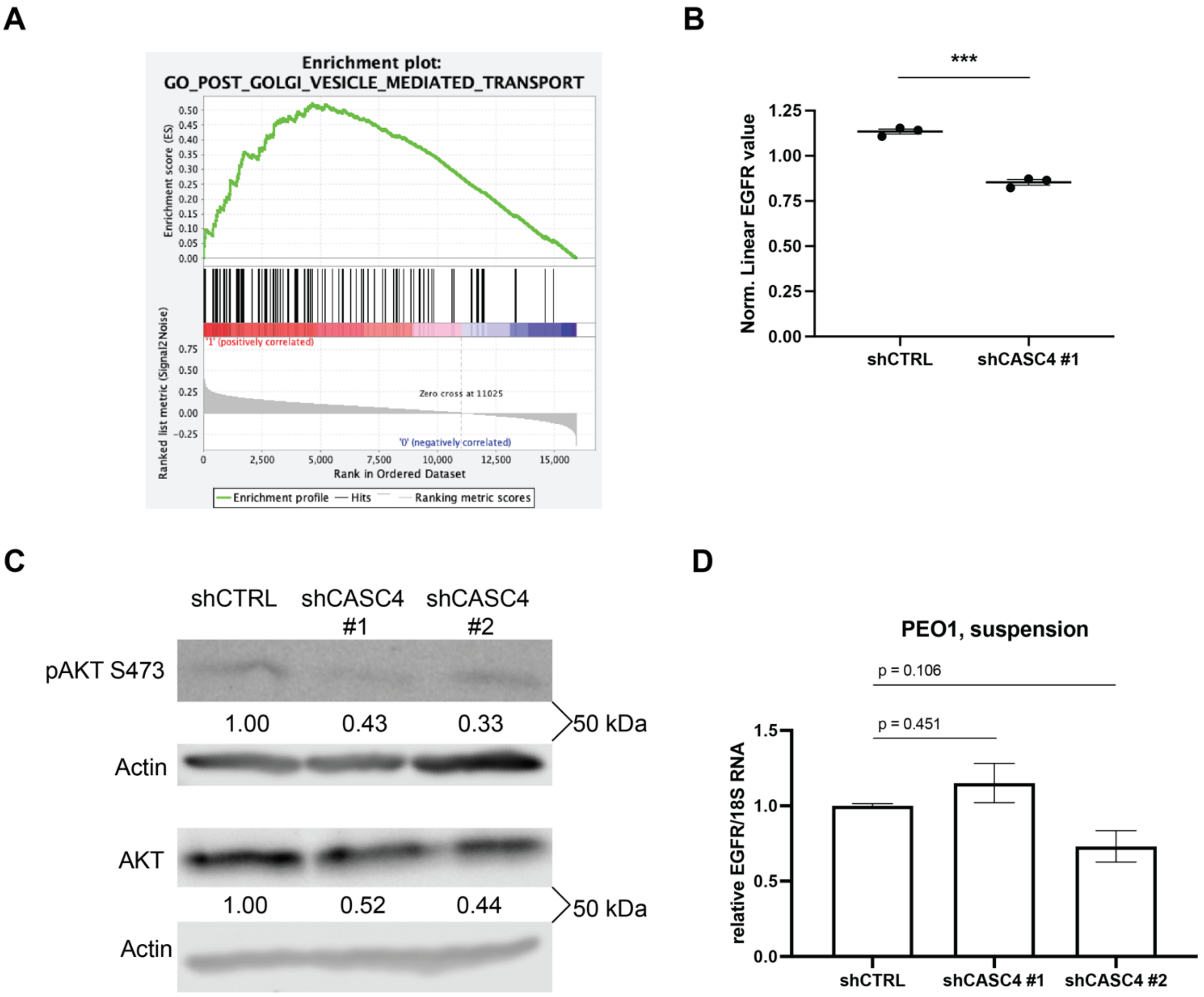

FIGURE S3. CASC4 KD leads to decreased EGFR protein levels. (A) GSEA enrichment plot performed on ovarian cancer patient RNA-seq data from (30). NES = 1.9. nominal p value < 0.001. q value = 0.02. FWER p value = 0.255. (B) EGFR Normalized Linear values as obtained from the RPPA plotted for PEO1 shCTRL and shCASC4 cells cultured in suspension. (C) Immunoblots showing pAKT (S473), AKT, and Actin levels in PEO1 shCTRL and shCASC4 cells cultured in suspension. Densitometries indicate fold change in pAKT or AKT relative to actin, and relative to shCTRL. Loading control, actin. (D) qRT-PCR experiments show that there is no significant difference in EGFR mRNA levels between PEO1 shCTRL and shCASC4 cells cultured in suspension. Values represent the mean ± SEM of 3 independent experiments. Statistical tests: (B) unpaired t-test; (D) one-way ANOVA. *p < 0.05, **p < 0.01, ***p < 0.001, **** p < 0.0001. Error bars show the SEM.

Figure S4, Bapat J, et al, 2022

A

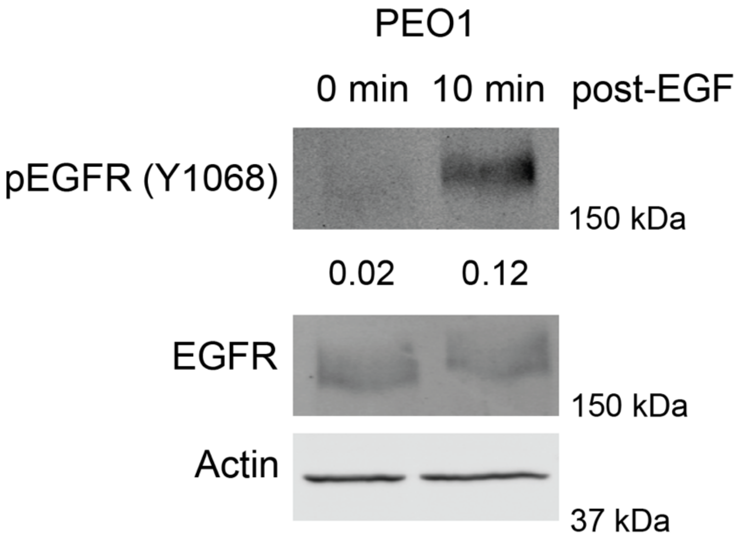

FIGURE S4. Activation of EGFR following EGF treatment. (A) Immunoblot showing EGFR and pEGFR protein levels 10 minutes after EGF treatment in PEO1 cells. Densitometries show fold change in pEGFR relative to EGFR. Loading control, actin.

A

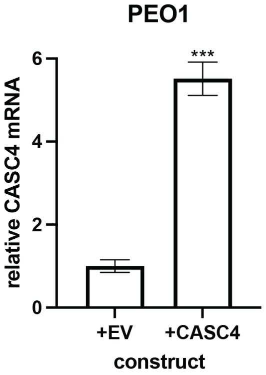

B

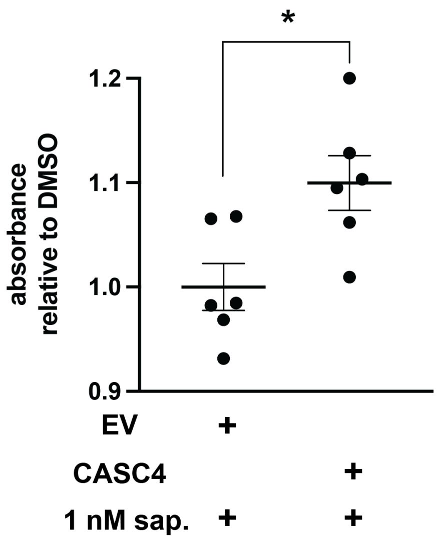

FIGURE S5. CASC4 overexpressing cells are more resistant to EGFR inhibition. (A) Validation of CASC4 overexpression in the PEO1 cell line by qRT-PCR. (B) Colony formation assays performed with CASC4 overexpressing cells. Cells were cultured in suspension for 2 days, with vehicle (DMSO) or 1 nM of sapitinib, then transferred to an adherent plate to allow for colony formation. Wells were stained with crystal violet, and the absorbances were measured and normalized, relative to DMSO treated cells and to the empty vector (EV) cells. Statistical tests: (A) unpaired t-test; (B) one-way ANOVA. *p < 0.05, **p < 0.01, ***p < 0.001, **** p < 0.0001. Error bars show the SEM.

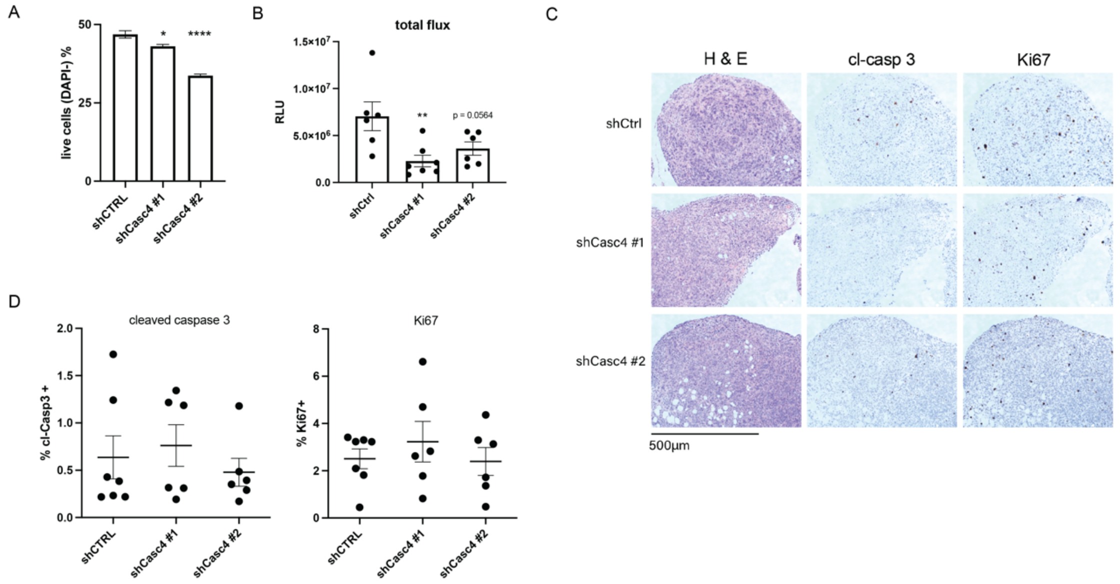

FIGURE S6. Murine Casc4 recapitulates human CASC4 phenotypes *in vitro*. (A) Flow cytometry was performed on ID8 cells expressing shCTRL or shCasc4 cultured in suspension for 2 days, gating on the DAPI$^-$ (live) cells. Values represent the mean ± SEM of 3 independent experiments. (B) Total relative luminescence units (RLU) of each mouse on day 35, as measured through IVIS. (C) Representative images of hematoxylin and eosin (H&E), cleaved caspase 3, and Ki67 staining performed on omentum from different mouse groups. (D) Quantification of percent cleaved-Caspase 3$^+$ and Ki67$^+$ cells in omentum tissue. Statistical tests: (A, B, D) one-way ANOVA. *p < 0.05, **p < 0.01, ***p < 0.001, **** p < 0.0001. Error bars show the SEM.

TABLE S1. List of Normalized Linear and Normalized Log2 Transformed Median Centered Z-scores from the reverse phase protein array analysis (RPPA).

TABLE S2. List of antibodies used for experiments.

Table S1

Target	normalized linear z-scores						normalized log 2 median centered scores					
	PEO1 shCTRL			PEO1 shCASC4			PEO1 shCTRL			PEO1 shCASC4		
14-3-3-beta-R-V	1.0681	0.9999	0.9273	0.9472	0.9663	0.9748	0.1382	0.0429	-0.0658	-0.0351	-0.0064	0.0064
14-3-3-epsilon-M-C	1.0154	1.0146	0.9689	0.9675	1.0077	0.9813	0.0302	0.0289	-0.0375	-0.0396	0.0192	-0.0192
14-3-3-zeta-R-V	1.1061	0.9833	0.9200	1.1426	0.9916	0.9864	0.1614	-0.0083	-0.1044	0.2083	0.0038	-0.0038
4E-BP1-R-V	1.0313	0.9571	0.9776	1.0217	1.0212	0.9832	0.0415	-0.0662	-0.0356	0.0280	0.0273	-0.0273
4E-BP1_pS65-R-V	1.0296	0.9559	0.9722	1.1015	1.0226	0.9973	0.0279	-0.0792	-0.0548	0.1253	0.0181	-0.0181
4E-BP1_pT37_T46-R-V	1.0422	0.9406	0.9715	1.0020	1.0399	0.9858	0.0686	-0.0795	-0.0328	0.0117	0.0653	-0.0117
53BP1-R-V	0.8439	0.8851	0.9390	1.0883	1.1024	1.1269	-0.2605	-0.1917	-0.1064	0.1064	0.1250	0.1568
A-Raf-R-V	0.9373	0.9422	0.9764	1.0688	1.0381	1.1087	-0.1032	-0.0957	-0.0442	0.0862	0.0442	0.1392
A-Raf_pS299-R-C	1.0214	1.0015	1.0019	0.9647	0.9999	0.9985	0.0295	0.0012	0.0017	-0.0529	-0.0012	-0.0032
ACC1-R-C	1.0759	1.0075	1.0023	1.0627	0.7414	0.7160	0.0985	0.0037	-0.0037	0.0807	-0.4388	-0.4891
ACC_pS79-R-V	0.8770	1.1029	1.2021	1.2589	0.9427	0.9062	-0.2173	0.1132	0.2375	0.3041	-0.1132	-0.1702
AceCS1-R-V	0.9982	1.0468	1.0665	0.9330	0.9014	0.9346	0.0475	0.1161	0.1430	-0.0499	-0.0997	-0.0475
ACLY_pS455-R-V	0.9507	1.0631	1.0111	1.2036	1.0774	0.9182	-0.1251	0.0362	-0.0362	0.2152	0.0555	-0.1752
ACSL1-R-V	0.8727	1.0128	1.0804	0.8949	1.0379	1.0797	-0.2324	-0.0177	0.0755	-0.1962	0.0177	0.0746
ACVRL1-R-C	1.1604	1.1273	1.1065	0.8737	0.8859	0.9085	0.2108	0.1691	0.1422	-0.1986	-0.1785	-0.1422
ADAR1-M-V	0.9756	1.0695	1.0375	1.0130	1.0299	1.0477	-0.0835	0.0491	0.0054	-0.0292	-0.0054	0.0194
Akt-R-V	0.9732	1.0934	1.0945	1.0818	0.9970	0.9879	-0.0937	0.0743	0.0757	0.0588	-0.0588	-0.0720
Akt1-R-V	0.9593	1.0723	1.0576	1.0312	1.0682	0.9922	-0.1225	0.0381	0.0182	-0.0182	0.0325	-0.0738
Akt1_pS473-R-V	1.0188	1.0551	1.0399	0.9139	0.9735	1.0142	0.0033	0.0538	0.0328	-0.1535	-0.0623	-0.0033
Akt2-R-V	0.9769	1.0522	1.0151	1.0854	1.0000	1.0463	-0.0772	0.0299	-0.0218	0.0747	-0.0435	0.0218
Akt2_pS474-R-C	1.0301	1.0324	1.0405	0.9273	0.9669	0.9600	0.0457	0.0489	0.0601	-0.1060	-0.0457	-0.0560
Akt_pS473-R-V	1.2265	1.1492	1.1806	0.8136	0.6533	0.6953	0.3430	0.2491	0.2880	-0.2491	-0.5657	-0.4757
Akt_pT308-R-V	0.9526	1.0728	1.1176	0.9230	0.9272	0.9106	0.0195	0.1910	0.2499	-0.0261	-0.0195	-0.0456
ALKBH5-R-V	1.0170	0.9384	0.8594	1.2225	1.0331	1.0394	-0.0113	-0.1274	-0.2542	0.2542	0.0113	0.0202
Ambra1_pS52-R-C	0.9844	0.9915	1.0122	0.9712	1.0358	1.0382	-0.0252	-0.0150	0.0150	-0.0447	0.0481	0.0515
AMPK-a2_pS345-R-V	1.0254	1.0292	1.0312	1.0077	0.9969	0.9941	0.0126	0.0179	0.0206	-0.0126	-0.0281	-0.0322
AMPKa-R-C	0.9690	0.9561	0.9822	1.0897	1.0577	0.9641	-0.0097	-0.0290	0.0097	0.1596	0.1166	-0.0171
AMPKa_pT172-R-C	0.7919	0.8283	0.6100	1.4135	1.2835	1.1633	-0.3098	-0.2450	-0.6863	0.5260	0.3869	0.2450
Annexin-I-M-V	1.0225	0.9557	1.0630	0.9270	0.5628	0.5396	0.1196	0.0220	0.1755	-0.0220	-0.7420	-0.8025
Annexin-VII-M-V	0.9343	0.8819	0.9350	1.2041	1.0951	1.1312	-0.1152	-0.1983	-0.1140	0.2508	0.1140	0.1608
AR-R-V	0.8957	0.9116	0.8404	1.0298	1.1041	1.0696	-0.1133	-0.0880	-0.2052	0.0880	0.1884	0.1427
ARID1A-R-C	1.0374	0.9785	1.0362	0.9800	0.9487	0.9127	0.0832	-0.0011	0.0816	0.0011	-0.0457	-0.1016
ASNS-R-V	1.0514	1.1460	1.0985	0.8802	0.7782	0.7780	0.1282	0.2525	0.1915	-0.1282	-0.3059	-0.3062
Atg3-R-V	1.0163	0.9737	1.0098	0.9285	0.9698	1.0040	0.0397	-0.0221	0.0304	-0.0907	-0.0278	0.0221
Atg4B-R-C	1.0053	0.9486	0.9258	1.0917	0.9914	1.0279	0.0101	-0.0736	-0.1088	0.1290	-0.0101	0.0422
Atg5-R-C	1.0146	0.9374	0.8974	1.0834	1.0434	1.0323	-0.0124	-0.1266	-0.1896	0.0822	0.0279	0.0124
Atg7-R-V	1.2217	1.1695	1.1452	0.8423	0.8091	0.9252	0.2472	0.1842	0.1539	-0.2893	-0.3473	-0.1539
ATM-R-V	0.9794	0.9263	0.8791	1.2166	1.0465	1.1002	-0.0478	-0.1283	-0.2037	0.2651	0.0478	0.1200
ATM_pS1981-R-V	0.9801	1.0389	1.0150	0.9403	0.9676	1.0428	-0.0252	0.0588	0.0252	-0.0851	-0.0437	0.0643
ATP5A-M-C	0.8293	1.3128	1.1529	1.0053	0.9917	0.6886	-0.2677	0.3948	0.2075	0.0098	-0.0098	-0.5360
ATP5H-R-V	1.0257	1.0505	1.0358	0.9807	0.9459	0.9507	0.0323	0.0668	0.0465	-0.0323	-0.0845	-0.0772
ATR-R-V	0.9746	0.9831	1.0187	1.0776	0.9909	1.0488	-0.0439	-0.0313	0.0200	0.1011	-0.0200	0.0620
ATRX-R-C	1.2648	1.1032	1.2716	0.7398	0.7353	0.7799	0.4474	0.2502	0.4551	-0.3263	-0.3352	-0.2502
ATR_pS428-R-C	1.0141	1.1427	1.0488	0.9835	0.8872	0.9481	0.0221	0.1944	0.0707	-0.0221	-0.1707	-0.0750
Aurora-A-R-C	0.9294	0.9113	0.9089	1.0264	1.0742	1.0699	-0.0716	-0.1000	-0.1038	0.0716	0.1372	0.1315
Aurora-ABC_pT288_pT232_pT198-R-C	1.0533	1.0343	0.9342	0.9439	0.9507	0.9787	0.1269	0.1006	-0.0463	-0.0314	-0.0210	0.0210
Aurora-B-R-V	1.0385	1.0458	1.0510	0.9188	0.8282	0.9441	0.0687	0.0788	0.0859	-0.1080	-0.2577	-0.0687
Axl-R-V	0.9911	0.9791	0.9378	1.1120	0.9781	1.0311	0.0088	-0.0088	-0.0709	0.1748	-0.0102	0.0658

b-Actin-R-C	1.1705	1.0616	0.9142	1.1436	0.8809	0.9009	0.2487	0.1078	-0.1078	0.2152	-0.1613	-0.1290
b-Catenin-R-V	0.9007	1.0086	0.8868	1.2243	1.1281	0.9702	-0.1352	0.0279	-0.1577	0.3076	0.1895	-0.0279
b-Catenin_pT41_S45-R-V	0.9582	0.9487	0.9578	0.9875	1.0694	0.9783	-0.0150	-0.0294	-0.0155	0.0284	0.1434	0.0150
B-Raf-R-C	0.8544	0.9870	0.9473	1.0991	1.1030	0.9953	-0.2142	-0.0060	-0.0653	0.1491	0.1542	0.0060
B-Raf_pS445-R-V	0.9677	0.9671	0.9473	1.2582	1.0398	1.0562	-0.0518	-0.0528	-0.0825	0.3268	0.0518	0.0743
B7-H3-R-C	1.0473	0.9636	0.9622	1.0163	0.9949	1.0111	0.0625	-0.0577	-0.0599	0.0190	-0.0116	0.0116
B7-H4-R-C	1.1440	1.0879	1.1010	0.8782	0.8793	0.9376	0.1798	0.1073	0.1245	-0.2017	-0.1998	-0.1073
Bad_pS112-R-V	0.9697	0.9777	0.9840	1.0633	1.0569	0.9347	-0.0164	-0.0046	0.0046	0.1164	0.1077	-0.0696
Bak-R-C	1.0268	0.9582	0.9767	1.0502	0.9279	1.0172	0.0429	-0.0568	-0.0293	0.0754	-0.1032	0.0293
BAP1-M-V	1.0053	0.9805	1.0063	1.0106	0.9779	1.0158	-0.0007	-0.0368	0.0007	0.0069	-0.0405	0.0144
Bax-R-V	0.9846	0.9735	1.0083	1.0150	1.0409	0.9409	-0.0171	-0.0335	0.0171	0.0267	0.0630	-0.0827
Bcl-xL-R-V	0.9687	1.0316	0.9068	1.2517	1.0580	0.9006	-0.0454	0.0454	-0.1407	0.3244	0.0819	-0.1505
Bcl2-R-C	1.0026	1.0389	1.0256	0.9861	0.9717	1.0187	-0.0115	0.0398	0.0213	-0.0355	-0.0566	0.0115
BCL2A1-R-V	1.0068	1.0196	1.1365	0.8488	0.9365	1.0142	-0.0053	0.0129	0.1696	-0.2515	-0.1097	0.0053
Beclin-R-C	0.9929	1.0622	1.0676	1.0169	1.0320	0.9704	-0.0450	0.0523	0.0597	-0.0106	0.0106	-0.0781
Bid-R-C	1.0186	0.9400	0.9490	1.0075	1.0199	1.0376	0.0079	-0.1079	-0.0941	-0.0079	0.0098	0.0346
Bim-R-V	0.9744	0.9507	0.9439	1.0005	1.0519	1.0418	-0.0191	-0.0546	-0.0649	0.0191	0.0913	0.0774
BiP-GRP78-M-C	1.0292	0.9669	1.0170	0.9660	0.9974	1.0074	0.0381	-0.0521	0.0209	-0.0533	-0.0072	0.0072
BMK1-Erk5_pT218_Y220-R-V	1.0589	0.9830	1.0074	0.9680	0.9314	0.9893	0.1028	-0.0046	0.0308	-0.0268	-0.0823	0.0046
BRCA1-M-C	1.0189	0.9819	0.9591	0.9491	1.0040	0.9660	0.0651	0.0118	-0.0222	-0.0373	0.0438	-0.0118
BRD4-R-V	0.9795	0.9918	1.0119	1.0145	1.0464	1.0407	-0.0488	-0.0307	-0.0018	0.0018	0.0465	0.0386
c-Abl-R-V	1.0042	0.9677	1.0383	1.0159	0.9813	1.0169	-0.0083	-0.0618	0.0398	0.0083	-0.0416	0.0098
c-Abl_pY412-R-C	0.9959	0.9998	1.0460	0.9485	0.9964	1.0259	-0.0031	0.0024	0.0676	-0.0735	-0.0024	0.0396
c-IAP2-R-C	0.9638	1.0004	1.0455	0.9904	0.9667	1.0604	-0.0465	0.0073	0.0709	-0.0073	-0.0421	0.0912
c-Jun_pS73-R-V	1.0333	1.0700	1.1264	0.8576	0.9398	0.9561	0.0560	0.1063	0.1805	-0.2129	-0.0807	-0.0560
c-Kit-R-V	1.0098	1.0394	1.0536	0.9214	0.8996	0.8667	0.0661	0.1078	0.1274	-0.0661	-0.1005	-0.1544
c-Met_pY1234_Y1235-R-V	1.0011	1.0197	1.0385	1.0135	0.9700	1.0203	-0.0222	0.0044	0.0307	-0.0044	-0.0677	0.0053
c-Myc-R-C	1.0062	0.9113	0.9504	0.9998	1.0334	1.0646	0.0046	-0.1383	-0.0776	-0.0046	0.0432	0.0861
C-Raf-R-C	0.9810	0.9932	1.0164	1.1584	1.0257	1.0419	-0.0578	-0.0398	-0.0066	0.1821	0.0066	0.0292
C-Raf_pS338-R-V	0.9467	0.4818	1.2352	0.9120	0.6413	1.1675	0.0270	-0.9475	0.4108	-0.0270	-0.5349	0.3293
CA9-R-C	0.9745	0.9161	0.9619	1.0253	1.0518	1.0589	-0.0367	-0.1258	-0.0555	0.0367	0.0734	0.0831
Calnexin-R-V	1.0197	0.9966	0.9592	1.0006	1.0031	1.0000	0.0277	-0.0054	-0.0605	0.0004	0.0041	-0.0004
Caspase-3-cleaved-R-C	1.0373	1.0143	0.9893	0.9354	0.9915	0.9439	0.0667	0.0344	-0.0016	-0.0824	0.0016	-0.0695
Caspase-7-cleaved--R-C	1.2503	1.4160	1.2592	0.7498	0.7050	0.8180	0.3060	0.4856	0.3163	-0.4316	-0.5205	-0.3060
Caspase-8-M-Q	0.7977	0.7769	0.8292	1.2306	1.2245	1.2769	-0.3372	-0.3752	-0.2812	0.2883	0.2812	0.3416
Caspase-8-cleaved-R-C	1.0160	1.0049	0.9837	0.9212	1.0084	0.9848	0.0304	0.0146	-0.0163	-0.1109	0.0195	-0.0146
Caveolin-1-R-V	0.8181	0.6794	0.7215	1.3234	1.2202	1.5861	-0.2884	-0.5563	-0.4696	0.4055	0.2884	0.6668
CD134-R-V	1.0289	0.9580	0.9444	0.9452	0.9085	1.0174	0.1127	0.0097	-0.0108	-0.0097	-0.0668	0.0965
CD171-M-V	0.9055	0.8237	0.8036	1.1441	1.1261	1.1746	-0.1573	-0.2939	-0.3295	0.1802	0.1573	0.2182
CD2-M-C	1.0631	0.9660	1.0085	0.8965	1.0217	1.0348	0.0667	-0.0715	-0.0094	-0.1792	0.0094	0.0277
CD20-R-C	0.9620	0.9442	0.8479	1.2311	1.0969	0.8319	0.0135	-0.0135	-0.1687	0.3693	0.2028	-0.1962
CD26-R-V	1.0339	1.0350	1.0266	0.9587	0.9230	0.9113	0.0597	0.0611	0.0493	-0.0493	-0.1042	-0.1225
CD29-M-V	0.9894	0.9524	0.9346	0.9366	1.0134	1.0328	0.0275	-0.0275	-0.0548	-0.0516	0.0620	0.0894
CD31-M-V	1.0145	0.9767	0.9702	0.8980	1.0016	1.0330	0.0366	-0.0181	-0.0279	-0.1394	0.0181	0.0627
CD38-R-C	0.9940	1.0019	1.0199	0.9178	1.0308	0.9989	-0.0093	0.0022	0.0278	-0.1244	0.0431	-0.0022
CD4-R-V	0.9911	0.9672	0.9911	0.9379	1.0340	1.0303	0.0000	-0.0352	0.0000	-0.0795	0.0611	0.0561
CD44-R-C	1.0658	0.9491	0.9256	1.0550	1.0302	1.0250	0.0526	-0.1146	-0.1509	0.0379	0.0037	-0.0037
CD45-M-V	1.0366	0.9656	0.9606	0.9539	0.9716	1.0089	0.0980	-0.0045	-0.0119	-0.0220	0.0045	0.0588
CD49b-M-V	0.9802	1.0815	1.0867	0.9397	0.9755	0.8979	0.0035	0.1454	0.1524	-0.0573	-0.0035	-0.1231
CD5-M-C	1.0051	0.9514	0.9711	0.9531	1.0277	1.0113	0.0249	-0.0545	-0.0249	-0.0519	0.0569	0.0337
CD68-M-C	1.0235	0.9749	1.0135	0.9426	1.0012	0.9829	0.0450	-0.0251	0.0309	-0.0737	0.0133	-0.0133
CD74-M-V	1.0100	0.9683	0.9253	0.9949	1.0136	1.0014	0.0170	-0.0438	-0.1093	-0.0047	0.0221	0.0047

cdc25C-R-V	1.0294	1.0064	1.0273	0.9638	0.9566	0.9899	0.0446	0.0119	0.0416	-0.0505	-0.0613	-0.0119
cdc2_pY15-R-C	1.0395	1.0149	1.0059	0.9512	0.9447	0.9790	0.0669	0.0324	0.0196	-0.0612	-0.0711	-0.0196
Cdc42-R-C	1.0674	1.0333	1.0469	0.9221	0.9624	0.9581	0.0981	0.0513	0.0702	-0.1130	-0.0513	-0.0577
Cdc6-R-V	1.0032	0.9623	0.9798	0.9391	1.0209	1.0066	0.0171	-0.0430	-0.0171	-0.0783	0.0422	0.0219
CDK1_pT14-R-C	0.9940	0.9275	0.9592	1.1896	1.0547	1.0384	-0.0315	-0.1315	-0.0829	0.2277	0.0539	0.0315
CDK9-R-V	0.9849	0.9495	0.9570	1.0106	1.0405	1.0218	-0.0185	-0.0713	-0.0600	0.0185	0.0607	0.0344
CDKN2A-R-C	1.0773	1.0564	0.9625	0.9664	0.8424	0.9521	0.1596	0.1313	-0.0029	0.0029	-0.1951	-0.0186
CDT1-R-V	1.0229	0.9800	1.0248	0.9177	1.0125	1.0023	0.0220	-0.0397	0.0248	-0.1345	0.0073	-0.0073
CENP-A-R-V	0.9741	0.9787	0.9301	0.9827	1.0523	0.9521	-0.0034	0.0034	-0.0701	0.0093	0.1079	-0.0363
cGAS-R-V	1.0117	1.0001	1.0422	0.8935	1.0010	1.0383	0.0077	-0.0090	0.0505	-0.1715	-0.0077	0.0451
CHD1L-R-V	1.0706	1.0726	1.0490	0.9203	0.8924	0.8371	0.1238	0.1265	0.0944	-0.0944	-0.1389	-0.2311
Chk1-M-C	0.8914	0.8879	0.8650	1.1443	1.0994	1.1323	-0.1513	-0.1570	-0.1947	0.2091	0.1513	0.1938
Chk1_pS296-R-V	1.0341	1.0041	1.0352	0.9316	0.9209	0.9414	0.0890	0.0465	0.0905	-0.0616	-0.0783	-0.0465
Chk1_pS345-R-C	0.9716	1.0751	1.0963	1.2534	0.9751	0.9007	-0.0756	0.0704	0.0985	0.2918	-0.0704	-0.1850
Chk2-M-V	0.8671	0.8723	0.8255	1.1074	1.1169	1.1409	-0.1808	-0.1722	-0.2517	0.1722	0.1845	0.2151
Chk2_pT68-R-C	1.0701	0.5399	1.0860	0.9495	0.6320	1.0531	0.0977	-0.8893	0.1191	-0.0747	-0.6621	0.0747
CIITA-R-C	0.9990	0.9862	1.0290	0.9603	1.0254	0.9813	0.0093	-0.0093	0.0520	-0.0477	0.0470	-0.0164
Claudin-7-R-V	0.9975	0.9210	1.0624	1.7466	1.0078	1.0590	-0.0506	-0.1656	0.0403	0.7576	-0.0357	0.0357
COG3-R-V	1.0383	1.0040	0.9803	0.9412	0.9183	0.9819	0.0817	0.0333	-0.0012	-0.0599	-0.0955	0.0012
Collagen-VI-R-V	1.0322	1.0532	1.0455	0.9232	0.9390	0.9285	0.0683	0.0973	0.0867	-0.0927	-0.0683	-0.0846
Complex-II-Subunit-M-V	0.9101	1.0365	1.0768	1.0697	1.0607	1.0374	-0.2049	-0.0173	0.0378	0.0281	0.0160	-0.0160
Connexin-43-R-C	1.0595	1.1527	0.9734	1.0593	0.8580	0.9576	0.0612	0.1828	-0.0610	0.0610	-0.2431	-0.0847
Coup-TFII-R-C	0.9672	0.9505	0.9659	1.0301	1.0051	1.0567	-0.0278	-0.0528	-0.0297	0.0632	0.0278	0.1000
Cox-IV-R-V	1.0359	1.0174	1.0244	0.9773	0.9248	0.9114	0.0549	0.0291	0.0389	-0.0291	-0.1087	-0.1298
Cox2-R-C	1.1233	1.1824	1.1154	0.8665	0.9743	0.9592	0.1077	0.1817	0.0975	-0.2667	-0.0975	-0.1201
CRABP1-R-C	1.0195	0.9879	1.0018	0.9568	0.9317	1.0006	0.0362	-0.0092	0.0110	-0.0553	-0.0937	0.0092
CRABP2-R-V	0.8739	0.8456	0.8513	1.3193	1.1471	1.2656	-0.1962	-0.2438	-0.2340	0.3980	0.1962	0.3380
Creb-R-C	1.0530	0.8378	1.0689	0.9894	0.8602	1.0696	0.0449	-0.2849	0.0665	-0.0449	-0.2468	0.0675
CREB_pS133-R-C	0.9967	1.0458	1.0682	0.9904	1.0274	0.9572	-0.0219	0.0474	0.0781	-0.0311	0.0219	-0.0802
CSK-R-C	0.8657	0.9827	0.9687	1.0371	1.1049	0.9934	-0.1906	-0.0079	-0.0286	0.0700	0.1612	0.0079
CtIP-R-V	0.6525	0.6369	0.6400	1.3005	1.3825	1.4065	-0.4975	-0.5324	-0.5254	0.4975	0.5857	0.6105
Cyclin-B1-R-V	1.1838	1.0446	0.9271	1.1543	0.9187	0.9199	0.2666	0.0861	-0.0861	0.2302	-0.0992	-0.0973
Cyclin-D1-R-C	0.9810	1.0008	1.0492	0.9411	1.0448	1.0150	-0.0391	-0.0102	0.0579	-0.0990	0.0519	0.0102
Cyclin-D3-M-V	0.9678	0.9130	0.9076	0.9644	1.0686	1.0561	0.0025	-0.0816	-0.0902	-0.0025	0.1454	0.1284
Cyclin-E1-R-V	1.0043	1.0093	1.0375	0.9335	0.9140	1.0169	-0.0036	0.0036	0.0433	-0.1091	-0.1395	0.0143
Cyclophilin-F-M-V	1.1136	1.0526	0.9384	1.0431	0.9135	0.9084	0.1706	0.0893	-0.0763	0.0763	-0.1150	-0.1231
D-a-Tubulin-R-V	1.0412	0.9701	1.0461	0.9244	0.9064	0.9964	0.0828	-0.0193	0.0896	-0.0888	-0.1173	0.0193
DAPK1_pS308-M-C	0.7580	1.1392	1.1319	1.2533	1.0119	0.6286	-0.4977	0.0901	0.0808	0.2278	-0.0808	-0.7677
DAPK2-R-C	0.9948	0.9963	1.0145	0.9648	0.9427	1.0271	-0.0011	0.0011	0.0272	-0.0453	-0.0787	0.0449
DDB-1-R-V	0.8824	1.0504	1.1151	1.0053	1.0281	0.9144	-0.2043	0.0471	0.1333	-0.0161	0.0161	-0.1529
DDR1-R-V	1.0442	1.0751	1.0515	0.9184	1.0061	0.9496	0.0268	0.0689	0.0368	-0.1585	-0.0268	-0.1103
DDR1_pY513-R-C	0.9209	1.0529	0.8969	0.9738	1.1095	0.9586	-0.0691	0.1240	-0.1073	0.0113	0.1996	-0.0113
DJ1-R-V	1.0036	0.9458	0.9589	1.2050	1.0340	0.9974	0.0044	-0.0811	-0.0613	0.2684	0.0475	-0.0044
DM-Histone-H3-R-V	1.0469	1.0342	1.0214	0.9427	0.9232	0.9326	0.0934	0.0757	0.0579	-0.0579	-0.0880	-0.0735
DM-K9-Histone-H3-M-V	1.0402	1.0411	1.0452	0.9234	0.9902	0.9667	0.0355	0.0367	0.0425	-0.1363	-0.0355	-0.0702
DNA-Ligase-IV-R-C	1.1536	1.1227	1.1410	0.8457	0.9028	0.9037	0.1957	0.1565	0.1799	-0.2522	-0.1580	-0.1565
DNA_POLG-R-V	0.8648	0.9421	0.9296	1.1662	1.0706	1.0352	-0.1916	-0.0680	-0.0873	0.2398	0.1165	0.0680
DNMT1-R-V	0.8549	0.8548	0.8503	1.3795	1.1678	1.1335	-0.2035	-0.2037	-0.2113	0.4869	0.2465	0.2035
DRP1-R-V	1.0423	1.0118	1.0362	0.9307	0.7944	0.8269	0.1030	0.0603	0.0946	-0.0603	-0.2888	-0.2309
DUSP4-R-V	0.9701	0.9365	0.9588	1.0391	0.9959	1.1178	-0.0189	-0.0698	-0.0359	0.0802	0.0189	0.1856
DUSP6-R-C	0.9080	0.9393	0.8636	1.0106	1.0413	1.0571	-0.1016	-0.0528	-0.1741	0.0528	0.0960	0.1177
Dvl3-R-V	0.9447	1.0333	1.0495	1.1308	1.0699	1.0283	-0.1406	-0.0113	0.0113	0.1189	0.0391	-0.0183

DYRK1B-R-C	0.9882	1.0064	0.9890	0.9200	0.9150	1.0342	-0.0006	0.0257	0.0006	-0.1037	-0.1116	0.0650
E-Cadherin-R-V	1.1242	1.0612	1.0311	0.9349	0.9135	0.9145	0.1954	0.1121	0.0707	-0.0707	-0.1041	-0.1024
E2F1-R-V	1.0132	0.9974	1.0251	0.9742	0.9726	1.0074	0.0154	-0.0072	0.0323	-0.0412	-0.0435	0.0072
eEF2-R-C	0.6567	1.0504	1.1580	1.0599	0.9868	0.6430	-0.6326	0.0450	0.1857	0.0581	-0.0450	-0.6630
eEF2K-R-V	0.9939	1.0434	1.0714	0.9252	0.9433	0.8618	0.0377	0.1078	0.1460	-0.0657	-0.0377	-0.1680
EGFR-R-V	1.1418	1.1536	1.1081	0.8724	0.8651	0.8235	0.2158	0.2305	0.1726	-0.1726	-0.1847	-0.2558
EGFR_pY1173-R-V	1.0464	1.0463	0.9935	1.0506	0.9726	0.9717	0.0375	0.0373	-0.0373	0.0432	-0.0680	-0.0694
eIF4E-R-V	1.0523	0.9207	0.8763	1.0578	1.0623	1.0133	0.0273	-0.1655	-0.2368	0.0348	0.0408	-0.0273
eIF4E_pS209-R-V	1.0467	0.9681	0.8865	1.0782	1.0088	0.9802	0.0739	-0.0388	-0.1658	0.1166	0.0208	-0.0208
eIF4G-R-C	0.9767	0.9624	0.9233	1.0301	1.0495	0.9976	-0.0153	-0.0364	-0.0963	0.0616	0.0885	0.0153
Elk1_pS383-R-C	1.1019	1.0863	0.9956	0.9637	0.8836	0.8893	0.1699	0.1493	0.0235	-0.0235	-0.1487	-0.1394
EMA-M-C	0.4197	0.4037	0.3747	1.5325	1.7694	1.6410	-0.9342	-0.9901	-1.0978	0.9342	1.1416	1.0329
Enolase-1-R-V	1.0566	0.9883	0.9970	1.2194	0.9860	0.9569	0.0900	-0.0064	0.0064	0.2968	-0.0097	-0.0529
Enolase-2-R-V	0.9553	0.9575	0.9236	0.9763	1.0343	1.0638	-0.0174	-0.0140	-0.0660	0.0140	0.0973	0.1378
ENY2-M-C	0.7321	1.0374	1.0973	1.0898	1.0452	0.6953	-0.5083	-0.0054	0.0756	0.0656	0.0054	-0.5826
EphA2-R-V	0.9533	0.9056	0.8874	1.0941	1.0747	1.2108	-0.0865	-0.1605	-0.1897	0.1123	0.0865	0.2585
EphA2_pS897-R-C	0.9056	0.8729	0.8993	1.1221	1.1243	1.1131	-0.1488	-0.2019	-0.1589	0.1604	0.1632	0.1488
EphA2_pY588-R-C	0.9922	1.0490	0.9362	0.9097	0.9266	1.0299	0.0419	0.1222	-0.0419	-0.0833	-0.0569	0.0956
ER-a-R-V	1.0326	1.0563	1.0042	1.0035	0.9509	0.9865	0.0407	0.0734	0.0005	-0.0005	-0.0782	-0.0252
ER-a_pS118-R-V	1.1236	0.9945	0.8308	1.2179	0.9790	0.9646	0.1873	0.0113	-0.2481	0.3037	-0.0113	-0.0328
ERCC1-R-C	0.9777	0.9292	0.9769	0.9991	1.0302	1.0512	-0.0156	-0.0889	-0.0168	0.0156	0.0599	0.0890
ERCC5-R-C	1.0394	1.0168	1.0204	1.0018	0.9782	0.9775	0.0425	0.0107	0.0159	-0.0107	-0.0451	-0.0461
Erk5-R-V	0.9903	0.9620	0.9288	0.9830	1.0348	1.0111	0.0053	-0.0365	-0.0872	-0.0053	0.0687	0.0354
ERRalpha-R-V	0.8993	0.9010	0.9258	1.0591	1.1342	1.0815	-0.1389	-0.1362	-0.0970	0.0970	0.1959	0.1273
Ets-1-R-V	0.9827	1.0657	1.0607	1.2382	1.0430	0.8644	-0.0981	0.0190	0.0121	0.2354	-0.0121	-0.2831
EVI1-R-V	0.8004	0.7778	0.7363	1.3101	1.2236	1.2755	-0.3061	-0.3475	-0.4266	0.4047	0.3061	0.3661
FABP5-R-C	1.0742	1.0542	0.9787	1.0067	0.9445	0.9173	0.1140	0.0869	-0.0204	0.0204	-0.0717	-0.1139
FAK-R-C	0.9348	1.0213	0.9875	1.2346	1.0945	0.9939	-0.1080	0.0196	-0.0290	0.2933	0.1194	-0.0196
FAK_pY397-R-V	1.1710	1.2201	1.1844	0.7914	0.7971	0.9587	0.1442	0.2036	0.1606	-0.4210	-0.4105	-0.1442
FANCD2-R-V	1.0389	1.0359	1.0272	0.9077	1.0244	1.0028	0.0183	0.0141	0.0019	-0.1765	-0.0019	-0.0327
FASN-R-V	1.0848	1.1315	1.0690	0.9042	0.7414	0.7387	0.1420	0.2028	0.1208	-0.1208	-0.4071	-0.4123
FGF-basic-R-C	0.6236	0.6780	0.6651	1.6886	1.4836	1.3324	-0.6080	-0.4874	-0.5150	0.8291	0.6424	0.4874
Fibronectin-R-V	1.8043	1.5826	1.6079	0.4670	0.3675	0.3770	1.0697	0.8805	0.9034	-0.8805	-1.2259	-1.1890
FN14-R-C	1.0489	1.0378	1.0466	0.9223	0.9552	0.8716	0.0752	0.0599	0.0720	-0.1104	-0.0599	-0.1920
FOXM1-R-V	1.1930	1.1018	1.0675	0.9053	0.8981	0.8832	0.2792	0.1644	0.1189	-0.1189	-0.1304	-0.1547
FOXO3-R-V	0.9186	0.8681	0.9298	1.0591	1.1120	1.1451	-0.1114	-0.1930	-0.0939	0.0939	0.1642	0.2066
FoxO3a_pS318_S321-R-C	0.9968	0.9579	1.0130	0.9742	0.9307	1.0249	0.0165	-0.0409	0.0397	-0.0165	-0.0825	0.0566
FRS2-alpha_pY196-R-V	0.9750	0.9999	1.0059	0.9450	0.9894	1.0484	-0.0288	0.0076	0.0162	-0.0739	-0.0076	0.0759
FTO-R-V	1.2548	1.1907	1.1490	0.8393	0.7996	0.8663	0.3307	0.2552	0.2037	-0.2495	-0.3192	-0.2037
G6PD-R-V	0.9195	0.9048	0.9120	1.0612	1.0802	1.1732	-0.1034	-0.1265	-0.1151	0.1034	0.1290	0.2482
Gab2-R-V	0.7406	0.7637	0.7586	1.2000	1.2394	1.2671	-0.3702	-0.3259	-0.3357	0.3259	0.3727	0.4045
GAPDH-M-C	0.9230	1.2625	1.2879	0.9335	0.7340	0.5184	-0.0081	0.4437	0.4724	0.0081	-0.3387	-0.8405
GATA3-M-V	1.0997	1.0805	1.0821	0.8935	0.9492	0.7963	0.1189	0.0934	0.0955	-0.1806	-0.0934	-0.3469
GATA6-R-V	1.0167	0.9880	1.0051	0.9330	0.9936	1.0035	0.0260	-0.0153	0.0094	-0.0979	-0.0072	0.0072
GCLM-R-V	1.0042	0.9916	0.9851	0.9908	0.9820	1.0170	0.0188	0.0005	-0.0088	-0.0005	-0.0134	0.0371
GCN5L2-R-V	0.9920	0.9978	0.9603	1.0907	1.0329	0.9790	-0.0042	0.0042	-0.0510	0.1327	0.0541	-0.0232
GGPS1-M-V	1.0284	1.0560	1.0327	0.9830	0.9489	0.8377	0.0326	0.0707	0.0386	-0.0326	-0.0836	-0.2634
Gli1-R-C	1.0029	0.9981	0.9767	0.9832	0.9149	1.0183	0.0178	0.0108	-0.0204	-0.0108	-0.1148	0.0398
Gli3-R-C	1.0106	1.0078	1.0017	0.9770	0.9892	1.0101	0.0084	0.0044	-0.0044	-0.0403	-0.0224	0.0078
Glucocorticoid-Receptor-R-V	0.8604	0.8068	0.8094	1.5439	1.1746	1.2421	-0.2246	-0.3173	-0.3128	0.6189	0.2246	0.3051
Glutamate-D1-2-R-V	0.7155	0.9372	1.0239	1.1299	1.0947	1.0408	-0.5288	-0.1394	-0.0118	0.1303	0.0846	0.0118
Glutaminase-R-C	0.8690	0.9030	0.8604	1.5437	1.2345	1.0793	-0.1841	-0.1287	-0.1984	0.6449	0.3225	0.1287

GRB2-R-V	0.9562	0.9310	0.9890	1.0598	1.0507	1.1526	-0.0924	-0.1308	-0.0436	0.0561	0.0436	0.1772
GRB7-R-V	1.0909	1.1179	1.1236	0.8598	0.8129	0.7864	0.1717	0.2070	0.2144	-0.1717	-0.2526	-0.3005
Grp75-R-C	0.9670	0.9219	0.8801	1.1137	1.0649	1.0569	-0.0642	-0.1330	-0.1999	0.1397	0.0750	0.0642
GSK-3a-b-M-V	0.9680	1.0609	1.0000	1.1191	1.0588	0.9896	-0.0882	0.0440	-0.0412	0.1211	0.0412	-0.0563
GSK-3a-b_pS21_S9-R-V	1.0070	1.0272	0.8505	1.1593	1.0168	0.9361	-0.0070	0.0216	-0.2506	0.1962	0.0070	-0.1123
GSK-3B-R-C	1.0505	1.0205	1.0188	0.9448	0.9113	0.9012	0.0986	0.0568	0.0544	-0.0544	-0.1065	-0.1226
Gys-R-V	0.9685	1.0298	0.9865	1.1684	1.0582	0.9833	-0.0575	0.0309	-0.0309	0.2132	0.0703	-0.0356
Gys_pS641-R-V	0.8868	1.0919	1.0442	1.1809	1.0963	0.9907	-0.2679	0.0322	-0.0322	0.1452	0.0380	-0.1081
H2AX_pS139-R-C	1.3062	1.2583	1.1658	0.8256	0.8394	0.8512	0.3909	0.3370	0.2269	-0.2710	-0.2470	-0.2269
H2AX_pS140-M-C	0.9970	1.0190	1.0184	1.0381	1.0276	0.8903	-0.0311	0.0004	-0.0004	0.0272	0.0125	-0.1943
HER2-M-V	0.9833	1.1145	1.0833	1.0204	1.0147	0.8710	-0.0495	0.1313	0.0902	0.0040	-0.0040	-0.2244
HER2_pY1248-R-V	0.9416	1.1089	0.9183	0.9449	1.0873	0.9891	-0.0380	0.1980	-0.0741	-0.0330	0.1695	0.0330
HER3-R-V	0.9792	0.9766	0.9381	0.9709	0.9475	1.0438	0.0080	0.0042	-0.0538	-0.0042	-0.0395	0.1003
HER3_pY1289-R-C	1.0247	1.1526	1.0369	1.1429	0.8578	0.8824	-0.0086	0.1612	0.0086	0.1489	-0.2650	-0.2243
Heregulin-R-V	0.8151	0.8900	0.9260	0.9941	1.0970	1.0958	-0.2351	-0.1084	-0.0512	0.0512	0.1933	0.1917
HES1-R-V	1.0918	1.0467	1.0285	0.9370	0.8757	0.8955	0.1533	0.0925	0.0672	-0.0672	-0.1648	-0.1325
Hexokinase-I-R-C	0.8764	0.9330	0.9404	1.0999	1.1539	1.0455	-0.1782	-0.0878	-0.0764	0.1496	0.2187	0.0764
Hexokinase-II-R-V	0.7616	0.7561	0.7812	1.5136	1.2480	1.2613	-0.3745	-0.3850	-0.3379	0.6164	0.3379	0.3533
Hif-1-alpha-R-C	1.0824	1.1219	1.1579	0.8320	0.9377	0.9169	0.1035	0.1552	0.2008	-0.2761	-0.1035	-0.1359
Histone-H3-R-V	1.1437	1.6612	1.7740	0.7238	0.8696	0.7114	0.1977	0.7362	0.8309	-0.4624	-0.1977	-0.4874
Histone-H3_pS10-R-V	1.0096	0.9448	0.8957	1.0020	1.0351	0.8030	0.0533	-0.0424	-0.1195	0.0424	0.0893	-0.2771
HLA-DQA1-R-V	0.9650	1.0232	1.0265	0.9662	0.9250	1.0590	-0.0431	0.0414	0.0460	-0.0414	-0.1043	0.0910
HLA-DR-DP-DQ-DX-R-C	0.9645	1.0239	1.0316	1.0493	1.0626	0.9729	-0.0916	-0.0054	0.0054	0.0300	0.0481	-0.0791
HMHA1-R-V	1.0624	0.9453	0.9560	1.0488	0.9679	1.0316	0.0884	-0.0800	-0.0638	0.0698	-0.0460	0.0460
HNRNPK-R-V	1.0081	1.0001	1.0343	0.9603	0.9395	1.0128	0.0057	-0.0057	0.0428	-0.0643	-0.0958	0.0126
HSP27-M-C	0.7733	0.7503	0.6892	1.2690	1.2546	1.2590	-0.3490	-0.3927	-0.5152	0.3655	0.3490	0.3541
HSP27_pS82-R-V	0.9646	0.8346	0.7858	1.1441	1.0891	1.0595	-0.0676	-0.2765	-0.3634	0.1785	0.1075	0.0676
HSP60-R-V	0.9765	0.9088	0.8919	1.1180	1.0496	1.0609	-0.0521	-0.1558	-0.1829	0.1431	0.0521	0.0675
HSP70-R-C	1.1622	1.0582	0.9428	1.0926	0.8993	0.9006	0.2185	0.0832	-0.0832	0.1295	-0.1515	-0.1493
IDO-R-C	1.0771	1.0986	1.0497	0.9175	1.0127	1.0006	0.0631	0.0915	0.0259	-0.1683	-0.0259	-0.0432
IGF1R_pY1135_Y1136-R-V	0.9986	0.9976	1.0038	0.9440	1.0259	1.0201	-0.0038	-0.0052	0.0038	-0.0849	0.0352	0.0270
IGFBP2-R-V	1.2769	1.0784	0.9766	0.9791	0.8772	0.8797	0.3850	0.1413	-0.0019	0.0019	-0.1566	-0.1526
IGFRb-R-C	0.9687	0.9105	0.9181	1.2317	1.0580	1.0842	-0.0636	-0.1531	-0.1411	0.2828	0.0636	0.0989
IL-6-R-C	0.9715	1.0050	0.9818	1.0000	1.0550	1.0350	-0.0453	0.0036	-0.0300	-0.0036	0.0736	0.0461
IMP3-R-C	1.0006	0.9585	0.9965	0.9333	0.9211	1.0208	0.0340	-0.0281	0.0281	-0.0665	-0.0855	0.0628
INPP4b-R-C	1.0328	1.0161	1.0278	0.9761	0.8336	0.8542	0.0525	0.0290	0.0455	-0.0290	-0.2566	-0.2215
IR-b-R-C	1.1022	1.0997	1.0943	0.8836	0.8659	0.9557	0.1081	0.1049	0.0977	-0.2108	-0.2400	-0.0977
IRF-1-R-V	1.3815	1.3716	1.3075	0.7106	0.7548	0.7412	0.4758	0.4654	0.3963	-0.4833	-0.3963	-0.4226
IRF-3-R-V	1.0005	0.9634	0.9780	1.0053	0.9488	1.0209	0.0164	-0.0381	-0.0164	0.0233	-0.0601	0.0455
IRS1-R-V	0.7954	0.7913	0.7190	1.2559	1.2083	1.2571	-0.3016	-0.3090	-0.4472	0.3573	0.3016	0.3587
IRS2-R-C	1.2959	1.2163	1.1259	0.8580	0.8272	0.8363	0.3989	0.3074	0.1960	-0.1960	-0.2487	-0.2330
JAB1-M-C	1.0300	0.9826	0.9455	0.9017	1.0315	0.9169	0.0958	0.0277	-0.0277	-0.0961	0.0978	-0.0721
Jagged1-R-V	1.0174	1.0146	0.9927	0.9643	0.9529	1.0028	0.0283	0.0243	-0.0073	-0.0491	-0.0663	0.0073
Jak2-R-V	1.0038	1.0125	0.9892	0.9931	1.0037	1.0174	0.0001	0.0125	-0.0211	-0.0154	-0.0001	0.0194
JNK2-R-V	0.8846	0.8850	1.1497	0.9047	1.0280	1.1746	-0.1246	-0.1239	0.2536	-0.0922	0.0922	0.2846
JNK_pT183_Y185-R-C	1.0009	1.0483	0.9919	1.0021	0.9161	1.0206	-0.0009	0.0659	-0.0138	0.0009	-0.1285	0.0272
KAP1-R-V	0.9690	0.9315	0.9528	1.0525	1.0501	1.0899	-0.0580	-0.1149	-0.0824	0.0613	0.0580	0.1116
KEAP1-R-V	0.9059	0.9654	0.9221	1.1928	1.0503	1.0478	-0.1509	-0.0591	-0.1253	0.2460	0.0625	0.0591
LAD1-R-V	1.0147	1.0123	1.1084	0.8715	0.9103	1.0316	0.0017	-0.0017	0.1291	-0.2179	-0.1550	0.0255
Lasu1-R-V	0.9729	1.0901	1.0949	1.1408	1.0132	0.8594	-0.1114	0.0528	0.0591	0.1183	-0.0528	-0.2902
LC3A-B-R-C	1.1499	1.1121	1.1024	0.8770	0.8287	0.8557	0.2258	0.1777	0.1650	-0.1650	-0.2467	-0.2004
Lck-R-V	0.9229	0.9458	0.9508	1.3090	1.0670	1.0312	-0.1015	-0.0662	-0.0586	0.4027	0.1077	0.0586

LCN2-R-V	1.8694	1.8915	0.0917	1.8179	0.0845	0.0766	2.1952	2.2121	-2.1548	2.1548	-2.2724	-2.4145
LDHA-R-C	0.8063	1.1222	1.3760	1.0394	0.8179	0.7059	-0.1935	0.2835	0.5776	0.1729	-0.1729	-0.3854
LRP6_pS1490-R-V	0.9591	0.9571	0.9551	1.0067	1.0156	1.0654	-0.0350	-0.0379	-0.0410	0.0350	0.0477	0.1167
Lyn-R-V	0.8895	0.9024	0.9681	1.0457	1.0830	1.1314	-0.1778	-0.1570	-0.0556	0.0556	0.1062	0.1693
MACC1-R-V	1.0342	0.9861	0.9474	1.0751	0.9148	0.9858	0.0689	0.0002	-0.0576	0.1249	-0.1081	-0.0002
MAPK_pT202_Y204-R-C	1.0080	0.9730	0.7236	1.1577	1.0157	0.8486	0.0255	-0.0255	-0.4527	0.2252	0.0365	-0.2229
Mcl-1-R-V	1.1333	1.0911	1.1599	0.8304	0.8859	0.8988	0.1946	0.1399	0.2281	-0.2540	-0.1607	-0.1399
MCT4-R-V	0.9722	1.0817	1.0416	1.1262	1.0543	0.9040	-0.1082	0.0458	-0.0087	0.1039	0.0087	-0.2131
MDM2_pS166-R-V	0.8384	0.9893	1.0236	1.2751	1.2655	0.9743	-0.2634	-0.0246	0.0246	0.3415	0.3306	-0.0466
MEK1-R-V	0.8980	0.8459	0.7107	1.3216	1.1643	1.1310	-0.1664	-0.2527	-0.5039	0.3910	0.2082	0.1664
MEK1_pS217_S221-R-V	0.9442	0.9345	0.9092	1.1748	1.0468	1.0544	-0.0743	-0.0893	-0.1289	0.2409	0.0743	0.0848
MEK2-R-V	0.9996	0.9825	0.9855	1.0115	1.0247	0.9850	0.0103	-0.0146	-0.0103	0.0273	0.0460	-0.0110
MelanA-R-C	0.9565	1.0306	1.0679	0.9535	1.0348	1.0633	-0.1106	-0.0029	0.0484	-0.1151	0.0029	0.0422
Melanoma-gp100-R-C	0.9793	0.9864	1.0267	0.9288	1.0351	1.0437	-0.0393	-0.0289	0.0289	-0.1157	0.0406	0.0526
MERIT40-R-C	0.9544	0.8959	0.9737	1.3328	1.0735	1.1107	-0.0993	-0.1905	-0.0704	0.3826	0.0704	0.1195
MERIT40_pS29-R-V	0.9666	0.9398	0.9591	1.0092	1.0737	1.0029	-0.0266	-0.0672	-0.0378	0.0356	0.1250	0.0266
Merlin-R-C	0.8419	1.0187	1.0824	1.0940	1.0595	0.7258	-0.3034	-0.0284	0.0592	0.0745	0.0284	-0.5174
METTL3-R-V	0.9542	0.9501	0.8880	1.1399	1.0794	1.0263	-0.0525	-0.0588	-0.1562	0.2040	0.1253	0.0525
Midkine-R-V	1.0432	1.0297	1.0143	0.9270	0.9116	0.9987	0.0517	0.0329	0.0112	-0.1186	-0.1429	-0.0112
MIF-R-C	1.0144	1.0564	1.0336	0.9480	0.9875	1.0061	0.0059	0.0645	0.0331	-0.0917	-0.0327	-0.0059
MIG6-R-V	1.0040	1.0348	1.0191	0.9563	0.9515	1.0172	-0.0095	0.0342	0.0121	-0.0797	-0.0870	0.0095
MITF-R-V	1.0347	0.9509	1.0091	0.9307	1.0282	0.9770	0.0595	-0.0624	0.0232	-0.0934	0.0504	-0.0232
Mitofusin-1-R-V	1.0439	1.0364	1.0138	0.9573	0.9282	0.9743	0.0709	0.0605	0.0286	-0.0540	-0.0986	-0.0286
Mitofusin-2-R-V	1.1567	1.0886	1.0245	0.9402	0.7616	0.7738	0.2370	0.1495	0.0619	-0.0619	-0.3660	-0.3430
MLH1-M-V	0.9672	0.9392	0.9006	0.9813	1.1428	1.0324	-0.0104	-0.0528	-0.1134	0.0104	0.2302	0.0837
MLKL-R-V	1.0429	0.9955	1.0042	0.9457	0.8999	0.9765	0.0809	0.0139	0.0263	-0.0601	-0.1318	-0.0139
MMP14-R-V	0.9652	0.9412	0.9810	1.2454	1.1297	1.0000	-0.0372	-0.0736	-0.0138	0.3305	0.1898	0.0138
MMP2-R-V	0.9963	1.0139	0.9679	0.9443	0.9427	1.0254	0.0208	0.0462	-0.0208	-0.0565	-0.0589	0.0624
Mnk1-R-V	0.9840	0.9501	0.9447	1.1043	1.0385	1.0387	-0.0389	-0.0895	-0.0976	0.1275	0.0389	0.0392
MR1-M-C	1.0553	1.0241	1.0414	0.9061	1.0263	0.9156	0.0418	-0.0016	0.0226	-0.1782	0.0016	-0.1632
MRAP-R-C	0.9796	1.0590	1.0495	0.9424	0.9307	1.0434	-0.0456	0.0670	0.0539	-0.1014	-0.1194	0.0456
MSH2-R-C	0.9482	1.0320	0.9652	1.3097	1.0801	0.9768	-0.0824	0.0396	-0.0569	0.3835	0.1054	-0.0396
MSH6-R-C	0.8300	0.9888	1.0792	1.2785	1.0626	0.8586	-0.3045	-0.0519	0.0743	0.3188	0.0519	-0.2556
MSI2-R-C	1.0572	1.0926	1.0517	0.9181	0.8485	0.9512	0.0799	0.1274	0.0724	-0.1235	-0.2373	-0.0724
MTCO1-M-V	1.1200	1.2165	1.1183	0.8641	0.8672	0.6059	0.1856	0.3049	0.1834	-0.1886	-0.1834	-0.7008
mTOR-R-V	1.1358	1.0157	0.9554	1.2008	0.8889	0.9525	0.2055	0.0441	-0.0441	0.2857	-0.1483	-0.0485
mTOR_pS2448-R-C	1.0533	1.0748	1.0049	1.2679	0.9350	0.8611	0.0339	0.0631	-0.0339	0.3015	-0.1380	-0.2567
MTSS1-M-C	0.9986	1.0006	1.0014	0.9320	1.0259	0.9981	-0.0015	0.0015	0.0027	-0.1010	0.0374	-0.0022
MYH11-R-C	0.9986	1.0136	1.0302	0.9844	1.0259	0.9191	-0.0108	0.0108	0.0342	-0.0314	0.0282	-0.1303
Myosin-IIa-R-C	0.9813	1.1111	1.1115	0.8806	0.9265	0.8329	0.0414	0.2207	0.2213	-0.1148	-0.0414	-0.1952
Myosin-IIa_pS1943-R-V	1.0593	1.1454	1.0164	0.9468	0.9607	0.6974	0.1003	0.2130	0.0407	-0.0618	-0.0407	-0.5028
Myt1-R-C	1.0171	1.0037	0.9789	1.0788	0.9780	1.0031	0.0196	0.0004	-0.0357	0.1045	-0.0369	-0.0004
N-Cadherin-R-V	0.9884	0.9784	1.0013	0.9594	0.9962	1.0339	-0.0056	-0.0204	0.0131	-0.0486	0.0056	0.0593
N-Ras-M-V	1.0038	0.9800	0.9716	0.9235	0.9604	1.0174	0.0407	0.0062	-0.0062	-0.0795	-0.0230	0.0602
NAPSIN-A-R-C	1.0198	0.9972	0.9855	0.9402	0.9772	1.0003	0.0408	0.0085	-0.0085	-0.0764	-0.0207	0.0129
NDRG1_pT346-R-V	0.8666	0.8531	0.7854	1.0075	1.1385	1.2821	-0.1087	-0.1313	-0.2506	0.1087	0.2851	0.4564
NDUFB4-R-V	1.0067	1.0105	1.0355	0.9316	0.9565	1.0142	-0.0027	0.0027	0.0380	-0.1146	-0.0765	0.0080
NF-kB-p65_pS536-R-C	1.0290	1.2327	1.0135	0.9904	0.9660	0.9904	0.0385	0.2991	0.0166	-0.0166	-0.0527	-0.0167
Notch1-R-V	0.9851	0.9866	0.9405	0.9690	0.9852	1.0375	-0.0001	0.0020	-0.0669	-0.0239	0.0001	0.0746
Notch1-cleaved-R-V	1.0061	0.9954	1.0209	0.9545	1.0075	1.0150	-0.0010	-0.0164	0.0201	-0.0769	0.0010	0.0117
Notch3-R-C	1.1726	1.1118	1.0844	0.8917	0.7860	0.7813	0.2540	0.1772	0.1411	-0.1411	-0.3231	-0.3317
NRF2-R-C	0.9712	1.0194	1.0377	0.9177	0.9646	1.0524	-0.0349	0.0349	0.0606	-0.1167	-0.0448	0.0809

Oct-4-R-C	0.7817	1.1392	0.8401	1.2175	0.9517	1.1462	-0.4136	0.1298	-0.3097	0.2256	-0.1298	0.1386
P-Cadherin-R-C	0.9776	0.9658	0.9613	1.0864	1.0485	0.9902	-0.0093	-0.0268	-0.0335	0.1429	0.0917	0.0093
p21-R-C	0.8678	0.8980	0.9519	0.9947	1.0880	1.1797	-0.1652	-0.1159	-0.0317	0.0317	0.1610	0.2778
p27-Kip1-R-C	1.0350	1.0259	1.0101	0.9682	0.9436	0.9839	0.0541	0.0414	0.0189	-0.0421	-0.0793	-0.0189
p27_pT157-R-C	0.9512	1.0155	1.0809	1.0243	1.0401	1.0580	-0.1178	-0.0235	0.0667	-0.0110	0.0110	0.0357
p27_pT198-R-V	0.9142	0.9660	1.0044	0.9839	1.0646	1.0335	-0.1209	-0.0414	0.0149	-0.0149	0.0989	0.0561
p38-a-M-V	0.9209	0.8662	0.9104	1.1844	1.1095	1.1457	-0.1345	-0.2228	-0.1509	0.2286	0.1345	0.1807
p38-MAPK-R-V	0.9650	0.9297	0.9380	1.1035	1.0521	1.0740	-0.0624	-0.1161	-0.1033	0.1311	0.0624	0.0920
p38-MAPK-_pT180_Y182-R-V	0.9928	0.9758	1.0116	0.9108	1.0321	0.8884	0.0125	-0.0125	0.0396	-0.1119	0.0685	-0.1479
p44-42-MAPK-R-V	0.9861	1.0475	1.0801	1.0285	0.9184	0.8889	-0.0303	0.0568	0.1010	0.0303	-0.1329	-0.1801
p53-R-C	1.6719	1.3043	1.2184	0.7830	0.7236	0.7541	0.7755	0.4173	0.3190	-0.3190	-0.4327	-0.3732
p70-S6K1-R-V	1.0064	0.9790	0.9606	1.1322	1.0028	1.0146	0.0026	-0.0372	-0.0646	0.1724	-0.0026	0.0143
p70-S6K_pT389-R-V	1.0258	1.0346	0.8700	1.1069	0.8685	0.9938	0.0228	0.0351	-0.2149	0.1327	-0.2172	-0.0228
p90RSK_pT573-R-C	1.1131	1.1405	1.1110	0.8701	0.9222	0.9434	0.1206	0.1557	0.1179	-0.2347	-0.1507	-0.1179
PAI-1-M-V	1.0014	1.0194	0.9272	0.9834	1.0228	1.0130	-0.0083	0.0174	-0.1193	-0.0345	0.0222	0.0083
PAICS-R-C	0.8157	0.8336	0.8094	1.4030	1.1605	1.1789	-0.2700	-0.2386	-0.2812	0.5124	0.2386	0.2613
PAK1-R-V	1.1640	1.1153	1.1491	0.8391	0.8101	0.8615	0.2479	0.1862	0.2293	-0.2242	-0.2750	-0.1862
PAK4-R-V	1.0199	0.9924	1.0423	0.9581	0.9795	0.9990	0.0346	-0.0048	0.0659	-0.0556	-0.0237	0.0048
PAK_pS474_S602_S560-R-V	1.0378	0.8718	0.7891	1.0761	1.1256	0.9653	0.0523	-0.1992	-0.3431	0.1045	0.1694	-0.0523
PAK_pT423_T402-R-C	0.9620	0.9946	0.9336	1.0302	1.0653	0.9369	-0.0241	0.0241	-0.0673	0.0748	0.1231	-0.0621
PAR-R-C	1.1812	1.2820	0.9689	0.9214	1.0082	0.9192	0.2572	0.3753	-0.0286	-0.1012	0.0286	-0.1046
PARG-R-C	0.9068	0.9165	0.9344	0.9565	1.0670	1.0651	-0.0600	-0.0447	-0.0169	0.0169	0.1746	0.1720
PARP-R-V	1.0010	1.0658	1.0634	0.9534	0.9232	0.8267	0.0351	0.1256	0.1224	-0.0351	-0.0817	-0.2409
Patched-R-C	1.0197	1.0055	0.9772	1.0287	0.9635	1.0004	0.0239	0.0037	-0.0374	0.0366	-0.0578	-0.0037
PAX6-R-V	1.0032	0.9841	0.9586	0.9558	1.0209	1.0152	0.0139	-0.0139	-0.0517	-0.0559	0.0392	0.0311
PAX8-R-C	1.0000	0.9472	0.9598	0.9969	0.9848	1.0295	0.0133	-0.0649	-0.0459	0.0088	-0.0088	0.0553
Paxillin-R-C	0.9976	0.9763	0.9770	1.0228	1.0185	1.0240	-0.0149	-0.0460	-0.0450	0.0210	0.0149	0.0228
PCNA-M-C	1.1212	0.9354	0.8550	1.1395	1.0457	0.9911	0.1393	-0.1222	-0.2518	0.1626	0.0388	-0.0388
PD-1-R-V	1.0679	1.0288	1.0242	0.9404	0.9248	0.9381	0.1218	0.0680	0.0616	-0.0616	-0.0858	-0.0651
PD-L1-R-C	1.0384	0.9771	1.0103	0.9904	0.9789	0.9959	0.0643	-0.0234	0.0247	-0.0039	-0.0208	0.0039
Pdcd4-R-C	1.2317	1.0963	1.1767	0.8168	0.8155	0.8474	0.3538	0.1857	0.2878	-0.2388	-0.2411	-0.1857
PDH-M-V	1.0062	0.9865	1.0138	0.9498	1.0000	1.0148	0.0045	-0.0241	0.0152	-0.0788	-0.0045	0.0167
PDHA1-R-V	1.0328	1.0548	1.0279	0.9385	0.9206	0.9474	0.0656	0.0960	0.0588	-0.0725	-0.1003	-0.0588
PDHK1-R-C	1.0453	1.0037	1.0172	0.9168	0.9589	1.0108	0.0534	-0.0051	0.0142	-0.1358	-0.0710	0.0051
PDK1-R-V	0.9496	0.9580	0.9803	1.3331	1.0423	1.0558	-0.0902	-0.0774	-0.0442	0.3992	0.0442	0.0628
PDK1_pS241-R-V	0.9251	0.9140	0.9569	1.2341	1.0699	1.1240	-0.1293	-0.1467	-0.0806	0.2866	0.0806	0.1517
PEA-15-R-V	0.9803	1.0527	0.9929	1.0758	1.0455	1.0027	-0.0628	0.0401	-0.0443	0.0714	0.0302	-0.0302
PEA-15_pS116-R-V	1.0808	0.9415	0.9695	0.9754	0.9484	1.0360	0.1524	-0.0467	-0.0044	0.0044	-0.0362	0.0913
PERK-R-V	0.9543	0.9777	0.9869	1.1288	1.0313	1.0668	-0.0802	-0.0453	-0.0317	0.1621	0.0317	0.0805
PGM1-R-V	1.0565	1.0244	0.9866	0.9973	0.9406	0.9608	0.0910	0.0465	-0.0078	0.0078	-0.0766	-0.0460
PHGDH-R-C	1.0419	1.0907	1.0921	0.8854	0.9302	0.9160	0.0818	0.1478	0.1497	-0.1530	-0.0818	-0.1040
PHLPP-R-V	1.2503	1.3538	1.4260	0.6364	0.6150	0.5505	0.4872	0.6018	0.6769	-0.4872	-0.5365	-0.6964
PI3K-p110-a-R-C	0.9202	1.0223	0.9382	1.1932	1.0407	1.0574	-0.1646	-0.0129	-0.1367	0.2102	0.0129	0.0359
PI3K-p110-b-M-C	0.9842	0.9979	0.9847	0.9849	1.0413	0.8461	-0.0009	0.0191	-0.0002	0.0002	0.0805	-0.2190
PI3K-p85-R-C	0.9796	1.0152	1.0796	0.9718	1.0463	1.0202	-0.0551	-0.0035	0.0852	-0.0667	0.0400	0.0035
PIP4K2A-R-V	0.9374	0.9239	0.9009	1.2203	1.0587	1.1308	-0.0878	-0.1087	-0.1450	0.2927	0.0878	0.1828
PIP4K2B-R-V	1.0505	1.0239	0.9917	0.9528	0.9061	0.9682	0.1005	0.0633	0.0173	-0.0405	-0.1129	-0.0173
PKA-a-R-V	1.0908	1.0585	1.0433	0.9250	0.9209	0.9445	0.1360	0.0926	0.0718	-0.1020	-0.1083	-0.0718
PKC-a-b-II_pT638_T641-R-V	1.0249	1.0272	1.0451	0.9236	0.9245	0.9759	0.0353	0.0386	0.0634	-0.1149	-0.1135	-0.0353
PKC-b-II_pS660-R-V	0.9673	0.9391	0.9885	1.1499	1.1037	1.0058	-0.0438	-0.0865	-0.0125	0.2057	0.1464	0.0125
PKC-delta_pS664-R-V	1.0018	0.9736	0.9878	0.9551	0.9903	1.0196	0.0184	-0.0226	-0.0018	-0.0504	0.0018	0.0439
PKCa-R-V	1.1712	1.1239	1.0716	0.9020	0.8808	0.9157	0.2416	0.1821	0.1134	-0.1352	-0.1695	-0.1134

PKM2-R-C	0.8003	0.8676	0.8495	1.8249	1.3217	1.1191	-0.3002	-0.1836	-0.2140	0.8891	0.4237	0.1836
PLC-gamma1-R-V	1.0443	1.0508	1.0150	1.0884	0.8941	0.9631	0.0205	0.0295	-0.0205	0.0802	-0.2034	-0.0963
PLC-gamma1_pS1248-R-V	1.0113	1.0418	1.0221	1.1671	0.9717	1.0094	-0.0077	0.0352	0.0077	0.1991	-0.0652	-0.0104
PLC-gamma2_pY759-R-V	1.0082	0.9945	0.9523	1.0874	0.9609	1.0127	0.0099	-0.0099	-0.0724	0.1189	-0.0594	0.0163
PLK1-R-C	1.0294	0.9657	0.9527	1.1325	0.8991	1.0087	0.0607	-0.0315	-0.0510	0.1984	-0.1345	0.0315
PMS2-R-V	0.9052	0.9280	0.9108	1.1043	1.1109	1.0511	-0.1258	-0.0898	-0.1168	0.1610	0.1696	0.0898
Porin-M-V	0.9712	0.9895	1.0225	0.9772	1.0554	1.0248	-0.0507	-0.0237	0.0237	-0.0417	0.0693	0.0269
PR-R-V	0.9696	0.9978	1.0197	0.8912	0.9803	1.1571	-0.0286	0.0128	0.0441	-0.1502	-0.0128	0.2265
PRAS40-M-C	1.0588	1.0086	1.0242	0.9249	0.9881	1.0012	0.0754	0.0054	0.0275	-0.1196	-0.0242	-0.0054
PRC1_pT481-R-C	1.0191	0.9866	0.9753	0.9772	1.0038	0.9638	0.0536	0.0069	-0.0098	-0.0069	0.0317	-0.0269
PREX1-R-V	0.9724	1.0077	0.9714	0.9662	1.0541	1.0223	-0.0258	0.0258	-0.0271	-0.0349	0.0906	0.0464
PTEN-R-V	0.9335	0.9120	0.8672	1.3562	1.1372	1.0692	-0.0979	-0.1315	-0.2041	0.4410	0.1869	0.0979
PTPN12-R-V	0.8927	0.9461	0.9086	1.0274	1.0728	1.0308	-0.1433	-0.0594	-0.1178	0.0594	0.1219	0.0641
Puma-R-C	1.0112	1.0067	1.0123	0.9697	1.0011	1.0094	0.0045	-0.0019	0.0060	-0.0559	-0.0099	0.0019
PYGB-R-V	0.9328	1.1832	1.1398	1.0818	0.9229	0.7812	-0.1069	0.2362	0.1823	0.1069	-0.1222	-0.3628
PYGM-M-C	1.0549	1.3359	1.1821	0.8137	0.7876	0.7591	0.1873	0.5279	0.3514	-0.1873	-0.2344	-0.2874
Pyk2_pY402-R-C	0.9884	1.1312	1.0775	0.8120	1.0027	1.1738	-0.0727	0.1220	0.0519	-0.3563	-0.0519	0.1754
Rab11-R-C	1.0123	1.0362	1.0186	1.0224	1.0111	0.9398	-0.0045	0.0292	0.0045	0.0098	-0.0061	-0.1117
Rab25-R-V	0.9536	1.0361	1.0490	1.3622	1.0534	1.0447	-0.1346	-0.0149	0.0030	0.3799	0.0090	-0.0030
Rad17_pS645-R-V	1.0950	1.0235	0.9902	0.9681	0.8758	0.8882	0.1615	0.0640	0.0163	-0.0163	-0.1608	-0.1406
Rad23A-R-C	0.9229	0.9815	0.9154	1.2695	1.1305	0.9889	-0.0943	-0.0054	-0.1060	0.3658	0.1985	0.0054
Rad50-R-V	1.0357	1.0248	1.0246	1.0577	0.8488	0.7887	0.0153	0.0001	-0.0001	0.0457	-0.2718	-0.3777
Rad51-R-C	0.9639	0.8811	0.8797	1.1107	1.0632	1.0881	-0.0707	-0.2002	-0.2026	0.1338	0.0707	0.1041
Raptor-R-V	0.9964	1.0988	1.0686	1.2000	1.0252	0.9642	-0.0710	0.0702	0.0299	0.1973	-0.0299	-0.1184
Rb-M-Q	0.8431	0.8280	0.7774	1.2169	1.1935	1.1637	-0.2324	-0.2586	-0.3495	0.2969	0.2690	0.2324
RBM15-R-V	0.9004	1.0699	0.9991	1.1657	1.0724	1.0258	-0.2184	0.0303	-0.0683	0.1541	0.0337	-0.0303
Rb_pS807_S811-R-V	1.2495	1.2171	1.0895	0.8875	0.8177	0.6149	0.3456	0.3078	0.1480	-0.1480	-0.2660	-0.6773
Rheb-M-C	0.9637	1.0337	1.0835	1.0503	1.0585	0.9899	-0.1127	-0.0115	0.0563	0.0115	0.0227	-0.0741
Rictor-R-C	1.0589	1.0188	0.9588	1.0582	0.9609	0.9484	0.0979	0.0422	-0.0454	0.0969	-0.0422	-0.0611
Rictor_pT1135-R-V	0.9983	1.1050	1.0664	1.0791	0.9958	0.9382	-0.0476	0.0989	0.0476	0.0647	-0.0512	-0.1373
RIP-R-C	1.0565	1.0762	1.0882	0.8813	0.9923	1.0510	0.0038	0.0304	0.0463	-0.2579	-0.0867	-0.0038
RIP3-R-C	1.0113	0.9769	1.0115	0.9205	0.9570	1.0094	0.0264	-0.0236	0.0266	-0.1094	-0.0533	0.0236
RPA32-R-V	0.9917	1.0475	1.0338	1.1757	1.0333	0.9782	-0.0597	0.0193	0.0004	0.1859	-0.0004	-0.0794
RPA32_pS4_S8-R-C	0.8761	0.9938	1.1003	1.1948	1.0423	0.6913	-0.2162	-0.0344	0.1124	0.2313	0.0344	-0.5580
RRM1-R-C	1.0249	0.9726	0.9809	0.9959	0.9801	1.0009	0.0523	-0.0232	-0.0109	0.0109	-0.0122	0.0182
RRM2-R-C	0.9533	1.0211	1.0403	1.0657	1.0746	0.9694	-0.1125	-0.0134	0.0134	0.0482	0.0602	-0.0884
RSK-R-C	1.0195	1.0179	0.9903	1.1481	0.9675	1.0006	0.0145	0.0124	-0.0274	0.1859	-0.0609	-0.0124
RSK1-R-V	1.0176	0.9908	1.0014	1.1368	0.9825	1.0026	0.0222	-0.0162	-0.0009	0.1821	-0.0283	0.0009
S100A4-R-V	1.0567	0.9942	1.0227	0.9282	1.0000	0.9668	0.0838	-0.0042	0.0365	-0.1032	0.0042	-0.0445
S6-M-V	0.9895	0.9774	0.9615	1.1041	1.0357	0.9561	0.0089	-0.0089	-0.0325	0.1670	0.0747	-0.0406
S6_pS235_S236-R-V	0.9474	0.9238	0.9677	1.3381	1.1879	1.0558	-0.0935	-0.1298	-0.0629	0.4047	0.2329	0.0629
S6_pS240_S244-R-V	0.9444	0.9839	1.0787	1.1967	1.0631	0.9920	-0.1208	-0.0618	0.0709	0.2207	0.0499	-0.0499
SCD-M-V	0.9763	0.9559	0.9600	0.9907	1.0499	1.0359	-0.0106	-0.0410	-0.0348	0.0106	0.0943	0.0750
SDHA-R-V	0.9735	1.0477	1.0942	0.9766	0.9740	0.9993	-0.0212	0.0848	0.1475	-0.0166	-0.0204	0.0166
SF2-R-V	1.0063	1.0000	0.9778	0.9159	0.9554	1.0147	0.0252	0.0162	-0.0162	-0.1105	-0.0496	0.0373
SFRP1-R-V	0.9534	0.9036	0.8858	1.2079	1.0746	1.1609	-0.0863	-0.1637	-0.1923	0.2550	0.0863	0.1979
SGK1-R-V	0.9755	0.9834	0.9674	1.0243	1.0507	1.0032	-0.0261	-0.0144	-0.0380	0.0443	0.0811	0.0144
SGK3-R-V	0.9532	0.8869	0.9485	0.9975	1.0858	1.0718	-0.0328	-0.1368	-0.0399	0.0328	0.1551	0.1364
Shc_pY317-R-C	0.9199	0.9534	1.3459	0.7788	1.0255	1.2912	-0.1041	-0.0526	0.4449	-0.3443	0.0526	0.3850
SHP-2_pY542-R-C	0.8358	1.0687	0.9318	0.9902	1.0718	1.0264	-0.2704	0.0842	-0.1136	-0.0259	0.0884	0.0259
SHP2-R-V	0.9124	0.9269	0.9739	1.2263	1.0553	1.0630	-0.1521	-0.1293	-0.0579	0.2745	0.0579	0.0683
SIRP-alpha-R-V	1.0432	0.9653	0.9164	0.9091	1.0227	0.9627	0.1139	0.0019	-0.0731	-0.0846	0.0853	-0.0019

SLC1A5-R-C	0.9230	0.9699	0.9291	1.1660	1.1161	1.0040	-0.0964	-0.0250	-0.0869	0.2407	0.1776	0.0250
Slfn11-G-C	0.9706	1.0437	1.0200	1.1307	1.0560	0.8199	-0.0882	0.0165	-0.0165	0.1321	0.0334	-0.3316
Smac-M-Q	0.8414	0.8185	0.7667	1.2973	1.1873	1.1744	-0.2405	-0.2804	-0.3747	0.3841	0.2562	0.2405
Smad1-R-V	1.0278	1.0511	1.0093	1.0728	0.9944	0.9428	0.0131	0.0456	-0.0131	0.0749	-0.0344	-0.1114
Smad3-R-V	0.9974	1.0600	1.0311	1.1995	1.0272	1.0095	-0.0452	0.0426	0.0028	0.2210	-0.0028	-0.0278
Smad4-R-V	1.1052	1.0376	1.1760	0.8173	0.6790	0.8001	0.2633	0.1721	0.3528	-0.1721	-0.4395	-0.2029
Snail-M-Q	0.9851	0.9677	1.0062	0.9215	0.9896	1.0375	-0.0033	-0.0290	0.0274	-0.0995	0.0033	0.0715
SOD1-M-V	0.7944	0.7759	0.7597	1.3886	1.2256	1.2756	-0.3128	-0.3467	-0.3772	0.4929	0.3128	0.3705
SOD2-R-V	1.0148	0.9940	0.9850	1.0134	1.0084	0.9984	0.0164	-0.0135	-0.0267	0.0143	0.0071	-0.0071
Sox17-R-V	0.9973	1.0192	1.0128	0.9717	0.9897	1.0243	-0.0111	0.0201	0.0111	-0.0488	-0.0222	0.0274
Sox2-R-V	1.0145	1.0868	1.0483	1.0141	0.9440	0.9905	0.0003	0.0995	0.0475	-0.0003	-0.1036	-0.0343
SOX7-R-V	0.9708	0.9305	0.9135	1.1353	1.0513	1.1249	-0.0575	-0.1187	-0.1453	0.1683	0.0575	0.1551
Src-M-V	1.0762	1.0194	1.0817	0.8938	0.9894	1.0105	0.0846	0.0063	0.0919	-0.1833	-0.0368	-0.0063
Src_pY416-R-V	0.8596	0.8947	0.7541	1.0053	1.2257	1.0886	-0.1419	-0.0841	-0.3307	0.0841	0.3701	0.1989
Src_pY527-R-V	0.9541	0.9975	0.8594	1.2231	1.0154	1.0708	-0.0770	-0.0128	-0.2278	0.2813	0.0128	0.0894
Stat1_pY701-R-V	0.9785	1.0121	1.0512	0.9132	1.0475	1.0016	-0.0412	0.0076	0.0623	-0.1408	0.0571	-0.0076
Stat3-R-C	0.9720	1.0333	1.0116	1.2610	1.0545	0.9627	-0.0730	0.0153	-0.0153	0.3026	0.0447	-0.0868
Stat3_pY705-R-C	1.0013	1.0077	0.9624	0.9530	1.0229	0.9814	0.0145	0.0237	-0.0427	-0.0569	0.0452	-0.0145
Stat5a-R-V	0.9354	0.9611	0.8988	1.1687	1.0211	1.0769	-0.0829	-0.0437	-0.1405	0.2385	0.0437	0.1204
Stathmin-1-R-V	1.0557	0.9979	1.0427	0.9255	0.8691	0.9861	0.0898	0.0086	0.0719	-0.1001	-0.1908	-0.0086
STING-R-V	1.0567	1.0947	1.0341	0.9325	0.8254	0.7725	0.1059	0.1568	0.0746	-0.0746	-0.2506	-0.3461
Syk-M-V	1.0804	1.0468	1.0538	0.9164	0.9835	0.9996	0.0789	0.0333	0.0430	-0.1586	-0.0566	-0.0333
Synaptophysin-R-C	1.0447	0.9862	1.0272	0.9704	0.9028	0.9819	0.0863	0.0031	0.0619	-0.0202	-0.1244	-0.0031
Tau-M-C	0.7483	0.6910	0.6984	1.3395	1.2989	1.2917	-0.3938	-0.5088	-0.4933	0.4462	0.4018	0.3938
TAZ-R-V	0.9481	1.3494	1.0287	1.2402	0.9560	1.0772	-0.1510	0.3582	-0.0332	0.2365	-0.1390	0.0332
TEAD-R-C	0.9927	1.1129	1.0949	0.8832	0.8788	0.8722	0.0843	0.2492	0.2256	-0.0843	-0.0915	-0.1023
TFAM-R-V	0.9616	0.9349	0.9551	1.0198	1.0462	1.0866	-0.0423	-0.0830	-0.0522	0.0423	0.0793	0.1339
TFRC-R-V	0.7426	0.9198	0.9515	1.4625	1.3141	1.0604	-0.4358	-0.1270	-0.0781	0.5420	0.3876	0.0781
TIGAR-R-V	1.0511	1.0352	1.0073	1.0380	0.9598	0.9014	0.0417	0.0197	-0.0197	0.0236	-0.0894	-0.1800
Transglutaminase-M-V	0.4973	0.6627	1.0865	1.5047	1.4475	1.0527	-1.1046	-0.6905	0.0228	0.4926	0.4367	-0.0228
TRAP1-M-V	0.9523	0.9561	0.8629	1.0874	1.0570	1.0412	-0.0672	-0.0614	-0.2096	0.1241	0.0832	0.0614
TRIM24-R-C	1.0598	0.9595	1.0125	1.0248	0.9344	1.0156	0.0637	-0.0797	-0.0023	0.0153	-0.1180	0.0023
TRIM25-R-C	1.0298	0.9938	0.9820	1.2053	0.9314	0.9895	0.0544	0.0032	-0.0141	0.2814	-0.0905	-0.0032
TRIP13-R-V	0.8662	1.0033	0.9752	1.1582	1.1254	0.9729	-0.1914	0.0205	-0.0205	0.2276	0.1862	-0.0239
TSC1-R-C	1.0699	0.9991	0.9410	1.1036	0.9738	0.9443	0.1172	0.0185	-0.0680	0.1620	-0.0185	-0.0629
TTF1-R-V	0.9952	1.0353	0.9996	1.0196	1.0295	0.9379	-0.0206	0.0364	-0.0143	0.0143	0.0283	-0.1062
Tuberin-R-V	0.9589	0.9546	0.9001	1.1107	1.0017	1.0657	-0.0315	-0.0381	-0.1229	0.1805	0.0315	0.1208
Tuberin_pT1462-R-V	1.0114	1.0257	1.0066	1.0184	0.9864	1.0092	0.0016	0.0218	-0.0053	0.0114	-0.0346	-0.0016
TUFM-R-V	0.9921	1.0140	1.0734	1.0010	0.9822	1.0192	-0.0223	0.0093	0.0915	-0.0093	-0.0366	0.0166
Twist-M-C	1.0056	0.9818	0.9931	0.9410	1.0183	0.9805	0.0263	-0.0083	0.0083	-0.0695	0.0444	-0.0101
Tyro3-R-V	0.9663	1.0582	1.1023	1.0688	0.9177	0.9061	-0.0655	0.0655	0.1244	0.0800	-0.1400	-0.1582
U-Histone-H2B-R-C	1.0561	1.0755	1.0018	0.9882	0.9156	0.8798	0.0861	0.1124	0.0098	-0.0098	-0.1199	-0.1774
UBAC1-R-V	1.0025	0.9654	0.9895	1.0022	1.0217	0.9902	0.0090	-0.0453	-0.0097	0.0087	0.0365	-0.0087
UBQLN4-M-C	0.9955	0.9593	0.9789	0.9521	0.9944	1.0263	0.0130	-0.0405	-0.0113	-0.0514	0.0113	0.0568
UGT1A-M-V	1.0348	1.0445	0.9868	0.9478	0.9868	0.9482	0.0686	0.0821	0.0000	-0.0581	0.0000	-0.0575
ULK1_pS757-R-C	1.0332	1.0011	0.9864	0.9394	0.8827	0.9858	0.0673	0.0218	0.0004	-0.0701	-0.1599	-0.0004
UQCRC2-M-C	0.9744	1.1429	1.0932	1.2019	0.9860	0.8759	-0.0915	0.1385	0.0744	0.2112	-0.0744	-0.2453
UVRAG-R-C	1.0369	1.0232	1.0111	0.9462	0.9549	0.9818	0.0576	0.0384	0.0212	-0.0745	-0.0614	-0.0212
VASP-R-V	1.0884	1.0112	0.9806	0.9734	0.8064	0.9575	0.1558	0.0497	0.0053	-0.0053	-0.2768	-0.0290
VAV1-R-C	0.9873	0.9200	0.9607	0.9474	0.9709	1.0784	0.0318	-0.0700	-0.0076	-0.0277	0.0076	0.1592
VEGFR-2-R-V	1.0506	1.0179	1.0363	0.9307	0.6800	0.6866	0.1103	0.0646	0.0905	-0.0646	-0.5173	-0.5033
VEGFR-2_pY1175-R-C	0.9376	1.1006	0.9761	0.9065	1.0258	1.0769	-0.0939	0.1374	-0.0358	-0.1425	0.0358	0.1060

VHL-R-C	0.9084	0.8856	0.8751	1.1137	1.1019	1.1463	-0.1393	-0.1759	-0.1932	0.1548	0.1393	0.1963
VHL-EPPK1-M-C	1.3166	1.0277	0.9785	1.1673	0.4580	0.7218	0.3927	0.0354	-0.0354	0.2191	-1.1306	-0.4745
Vinculin-M-V	0.9126	0.8507	0.8422	1.2047	1.1185	1.1984	-0.1468	-0.2481	-0.2626	0.2540	0.1468	0.2463
Wee1-R-C	1.0381	1.0202	1.0058	0.9901	0.9644	0.9805	0.0570	0.0319	0.0113	-0.0113	-0.0493	-0.0253
Wee1_pS642-R-C	1.0033	0.9996	0.9935	0.9918	1.0208	0.9691	0.0096	0.0044	-0.0044	-0.0070	0.0347	-0.0403
WIPI1-R-C	0.9101	1.1172	1.1266	1.1362	1.0170	0.8935	-0.2280	0.0678	0.0800	0.0921	-0.0678	-0.2545
WIPI2-R-C	1.0085	1.0225	1.0550	0.9465	0.9697	0.9876	0.0151	0.0350	0.0801	-0.0765	-0.0415	-0.0151
WTAP-R-V	0.8901	0.8890	0.8635	1.1946	1.1203	1.0950	-0.1495	-0.1513	-0.1932	0.2750	0.1824	0.1495
XBP-1-G-C	0.9758	1.0016	1.0502	0.9968	1.0504	0.9644	-0.0342	0.0034	0.0718	-0.0034	0.0721	-0.0512
XIAP-R-C	1.0074	1.0624	1.0563	1.0762	0.9585	0.9914	-0.0342	0.0426	0.0342	0.0612	-0.1060	-0.0573
XPA-M-V	1.1446	1.0784	1.1049	0.8750	0.9058	0.8449	0.2119	0.1258	0.1609	-0.1757	-0.1258	-0.2262
XPF-R-C	0.9689	0.9472	0.9309	1.0084	0.9482	1.0549	0.0155	-0.0171	-0.0422	0.0732	-0.0155	0.1382
XRCC1-R-C	1.0237	0.9705	0.9821	1.0278	0.9684	1.0033	0.0445	-0.0324	-0.0154	0.0503	-0.0356	0.0154
YAP-R-C	1.0297	0.9943	1.0292	0.9707	0.9300	0.9896	0.0539	0.0034	0.0532	-0.0312	-0.0931	-0.0034
YAP_pS127-R-V	0.9830	0.9520	0.9674	1.0141	0.9952	1.0398	-0.0089	-0.0551	-0.0320	0.0361	0.0089	0.0721
YB1_pS102-R-V	1.0931	1.0627	0.9147	1.0690	0.9241	0.8894	0.1415	0.1008	-0.1156	0.1094	-0.1008	-0.1560
YES1-R-V	0.9915	1.0088	1.0109	0.9690	0.9932	1.0306	-0.0138	0.0112	0.0142	-0.0468	-0.0112	0.0421
YTHDF2-R-V	1.1741	0.9904	0.9521	1.2775	0.9481	0.9810	0.2524	0.0069	-0.0500	0.3741	-0.0560	-0.0069
YTHDF3-R-C	0.9199	0.8997	0.8476	1.3315	1.0860	1.1513	-0.1197	-0.1518	-0.2378	0.4138	0.1197	0.2040
ZAP-70-R-V	1.0481	0.9474	0.9016	0.9990	0.8665	1.0294	0.1075	-0.0383	-0.1096	0.0383	-0.1669	0.0815
ZEB1-R-V	1.0186	0.9581	1.0352	0.9436	0.9564	1.0173	0.0451	-0.0432	0.0685	-0.0652	-0.0458	0.0432

TABLE S2

Antibody	Host species	Target species	Application	Dilution	Manufacturer	Catalog #
β-actin	Ms	Hu	IB	1:5000	Abcam	ab6276
AKT	Rb	Hu	IB	1:1000	Cell Signaling Technologies	9272
CASC4	Rb	Hu	IF	1:150 (IF)	Abcam	ab230522
cleaved caspase 3	Rb	Ms	IHC	1:500	Cell Signaling Technologies	9661
EGFR	Rb	Hu	IB	1:500	Cell Signaling Technologies	4267
EGFR	Rb	Hu	IB	See methods	Cell Signaling Technologies	54359
EGFR	Ms	Hu	IB, IF	1:1000	Abcam	ab289889
EGFR-AlexaFluor 488	Ms	Hu	FACS	1:800	BioLegend	352907
GM130	Ms	Hu	IF	1:100	BD Biosciences	610822
Ki-67	Rb	Ms	IHC	1:250	Thermo Scientific	RM-9106
pAKT (S473)	Rb	Hu	IB	1:1000	Cell Signaling Technologies	9271
pEGFR (Y1068)	Rb	Hu	IB	1:1000	Cell Signaling Technologies	3777
RAB11	Rb	Hu	IF	1:100	Cell Signaling Technologies	5589
Ubiquitin	Rb	Hu	IB	1:1000	Cell Signaling Technologies	43124
Anti-rabbit IRDye 680RD	Dk	Rb	WB	1:20000	LI-COR	926-68071
Anti-mouse IRDye 800CW	Dk	Ms	WB	1:20000	LI-COR	926-32210
Alexa Fluor® 488 AffiniPure Fab Fragment Donkey Anti-Rabbit IgG (H+L)	Dk	Rb	IF	1:100	Jackson ImmunoResearch	AB_2340620
Cy™3 AffiniPure Fab Fragment Donkey Anti-Mouse IgG (H+L)	Dk	Ms	IF	1:100	Jackson ImmunoResearch	AB_2340818

Dk = donkey;
Ms = mouse;
Hu = human;
Rb = rabbit;
FACS = flow cytometry;
IB = immunoblot;
IF = immunofluorescence;
IHC = immunohistochemistry;
IP = immunoprecipitation
